# The trichome pattern diversity of Cardamine shares genetic mechanisms with Arabidopsis but differs in environmental drivers

**DOI:** 10.1093/plphys/kiae213

**Published:** 2024-04-12

**Authors:** Alberto Fuster-Pons, Alba Murillo-Sánchez, Belén Méndez-Vigo, Arnald Marcer, Bjorn Pieper, Rafael Torres-Pérez, Juan Carlos Oliveros, Miltos Tsiantis, F Xavier Picó, Carlos Alonso-Blanco

**Affiliations:** Departamento de Genética Molecular de Plantas, Centro Nacional de Biotecnología (CNB), Consejo Superior de Investigaciones Científicas (CSIC), Madrid 28049, Spain; Departamento de Genética Molecular de Plantas, Centro Nacional de Biotecnología (CNB), Consejo Superior de Investigaciones Científicas (CSIC), Madrid 28049, Spain; Departamento de Genética Molecular de Plantas, Centro Nacional de Biotecnología (CNB), Consejo Superior de Investigaciones Científicas (CSIC), Madrid 28049, Spain; CREAF, Cerdanyola del Vallès 08193, Spain; Universitat Autònoma de Barcelona, Cerdanyola del Vallès 08193, Spain; Department of Comparative Development and Genetics, Max Planck Institute for Plant Breeding Research, Carl-von-Linné Weg 10, 50829 Cologne, Germany; Departamento de Genética Molecular de Plantas, Centro Nacional de Biotecnología (CNB), Consejo Superior de Investigaciones Científicas (CSIC), Madrid 28049, Spain; Departamento de Genética Molecular de Plantas, Centro Nacional de Biotecnología (CNB), Consejo Superior de Investigaciones Científicas (CSIC), Madrid 28049, Spain; Department of Comparative Development and Genetics, Max Planck Institute for Plant Breeding Research, Carl-von-Linné Weg 10, 50829 Cologne, Germany; Departamento de Biología evolutiva, Estación Biológica de Doñana (EBD), Consejo Superior de Investigaciones Cientíﬁcas (CSIC), Sevilla 41092, Spain; Departamento de Genética Molecular de Plantas, Centro Nacional de Biotecnología (CNB), Consejo Superior de Investigaciones Científicas (CSIC), Madrid 28049, Spain

## Abstract

Natural variation in trichome pattern (amount and distribution) is prominent among populations of many angiosperms. However, the degree of parallelism in the genetic mechanisms underlying this diversity and its environmental drivers in different species remain unclear. To address these questions, we analyzed the genomic and environmental bases of leaf trichome pattern diversity in *Cardamine hirsuta*, a relative of Arabidopsis (*Arabidopsis thaliana*). We characterized 123 wild accessions for their genomic diversity, leaf trichome patterns at different temperatures, and environmental adjustments. Nucleotide diversities and biogeographical distribution models identified two major genetic lineages with distinct demographic and adaptive histories. Additionally, *C. hirsuta* showed substantial variation in trichome pattern and plasticity to temperature. Trichome amount in *C. hirsuta* correlated positively with spring precipitation but negatively with temperature, which is opposite to climatic patterns in *A. thaliana*. Contrastingly, genetic analysis of *C. hirsuta* glabrous accessions indicated that, like for *A. thaliana*, glabrousness is caused by null mutations in *ChGLABRA1* (*ChGL1*). Phenotypic genome-wide association studies (GWAS) further identified a *ChGL1* haplogroup associated with low trichome density and *ChGL1* expression. Therefore, a *ChGL1* series of null and partial loss-of-function alleles accounts for the parallel evolution of leaf trichome pattern in *C. hirsuta* and *A. thaliana*. Finally, GWAS also detected other candidate genes (e.g. *ChETC3*, *ChCLE17*) that might affect trichome pattern. Accordingly, the evolution of this trait in *C. hirsuta* and *A. thaliana* shows partially conserved genetic mechanisms but is likely involved in adaptation to different environments.

## Teaser

Genomic and environmental approaches show that different environmental drivers led to similar genetic mechanisms accounting for the evolution of trichome pattern in Cardamine and Arabidopsis.

## Introduction

Understanding the mechanisms underlying the genetically based adaptation of traits, so-called evolutionary adaptations, has become a major goal in plant biology fueled by the current scenario of climate change (Franks and Hoffmann 2012; Lippmann et al. 2019; Langridge et al. 2021). A plethora of initiatives are now dissecting the genomic bases of highly heritable traits of wild and crop plants, such as flowering time, plant and organ architecture, or pathogen resistance (Alonso-Blanco and Méndez-Vigo 2014; Bailey-Serres et al. 2019; Gaudinier and Blackman 2020). However, highly variable and complex traits, such as the amount and distribution of trichomes in different plant organs, referred to as trichome pattern, have largely been neglected (Nicotra et al. 2010) despite their potential relevance for adaptation to multiple abiotic and biotic factors (Schaller 2008; Hauser 2014; Bickford 2016).

Trichomes, or plant hairs, are highly differentiated cells that grow in the surface of leaves and other organs of most angiosperm plants. They can be uni- or multicellular, and their morphology can vary from simple (unbranched or single stalk) to highly branched (Judd et al. 1999). The broad interspecific diversity of trichome pattern and morphology has classically been used as taxonomic traits in angiosperms (Tutin et al. 1993; Judd et al. 1999). In addition, substantial intraspecific variation has been described among populations, which is presumably involved in adaptation to numerous environmental stresses. These include protection from UV radiation and drought, as well as physical and chemical defenses against herbivore insects and pathogens (Schaller 2008; Fürstenberg-Hägg et al. 2013; Bickford 2016; Matsumura et al. 2022). Trichome pattern also varies along the ontogenic sequence of plants (developmental plasticity; Wang et al. 2019), and across different environments (environmental plasticity), as it responds to several stresses, although this has been mainly documented for herbivore attack (Scoville et al. 2011; Sobral et al. 2021). The evolution of trichome pattern is hypothesized to be constrained by ecological costs, such that a reduction of plant fitness is caused by trade-offs between plant defenses and growth or reproduction (Züst and Agrawal 2017). Accordingly, trichome pattern plasticity enables environmental acclimation and may reduce the costs associated with defense to the necessary period (Schaller 2008).

In the past decades, the genetic and molecular bases of trichome development have been elucidated mainly in the leaves of the major Brassicaceae model plant, Arabidopsis (*Arabidopsis thaliana*). This is characterized by unicellular and branched trichomes, and ∼150 genes have been identified affecting its trichome pattern and morphology. In particular, leaf trichome pattern appears as regulated by a core gene network involving a trimeric activating complex encoded by *GLABRA1* (*GL1*), *GLABRA3/ENHANCER OF GLABRA3* (*GL3*/*EGL3*), and *TRANSPARENT TESTA GLABRA1* (*TTG*), which triggers trichome initiation by promoting the expression of the homeodomain gene *GLABRA2* (*GL2*). In addition, seven R3 MYB transcription factors repress trichome development by disturbing the function of that trimeric complex (Wang and Chen 2014). Recent studies have investigated the ontogenic variation of trichome pattern (Wang et al. 2019), the development of trichomes in plant organs other than leaves (Sun et al. 2015), as well as the environmental and hormonal regulation of trichomes (Pattanaik et al. 2014; Fambrini and Pugliesi 2019). Furthermore, the interspecific diversity of trichomes is now addressed in diverse crops, such as tomato (*Solanum lycopersicum*) and cotton (*Gossypium hirsutum*), with glandular or fiber-like trichomes, respectively (Chalvin et al. 2020; Schuurink and Tissier 2020; Wang et al. 2022). These studies indicate that MYB, bHLH, C2H2 zinc finger, and homeodomain transcription factors show conserved functions in trichome development in angiosperms (Han et al. 2022).

Despite the increasing knowledge on the regulation of trichome pattern, only nine genes have been identified explaining the natural intraspecific diversity in different plants. Most studies have focused on the qualitative variation segregating in glabrous and hairy populations of wild and cultivated Brassicaceae species, demonstrating that this is caused by independent loss-of-function mutations in the R2R3 MYB transcription factor GL1 (Hauser et al. 2001; Kivimäki et al. 2007; Li et al. 2013; Xuan et al. 2020). In addition, three R3 MYB genes, *ENHANCER OF TRY AND CPC 2* (*ETC2*), *TRICHOMLESS1* (*TCL1*), and *TRIPTYCHON* (*TRY*), as well as *AtMYC1*, contribute to the quantitative variation in leaves, pedicels and fruits of *Arabidopis thaliana* (Hilscher et al. 2009; Symonds et al. 2011; Arteaga et al. 2021, 2022). Similarly, *PD1*, *Ps*, and *P1* genes of soybean, and *HAIRY* (*H*) in *Antirrhinum*, have been recently found to account for their intraspecific diversity (Liu et al. 2020; Tan et al. 2020). Further characterization of these genes has suggested that this natural variation is involved in adaptation to contrasting environments, since high trichome density has been associated with low precipitation or alpine habitats in *A. thaliana* and *Antirrhinum*, respectively, but with low drought stress in soybean (Liu et al. 2020; Tan et al. 2020; Arteaga et al. 2022). However, the conservation of such adaptions has not been addressed in other plants, with the remarkable exception of *Antirrhinum* genus (Tan et al. 2020).

To enable comparative evolutionary studies in different species, several model plants have been developed in Brassicaceae, as one of the most diverse angiosperm families (Schmidt and Bancroft 2011; Huang et al. 2016; Nikolov et al. 2019). Thus, convergent evolution, broadly defined as the independent evolution of similar phenotypic features in different plants, is a rather frequent process for numerous characters, including trichome traits (Stern 2013; Morris 2015; Huang et al. 2016). However, deciphering the precise function of convergent evolution in plant adaptation to similar environments, so-called convergent adaptation, requires comparative analyses of intraspecific diversity in different species (Rellstab et al. 2020; Bohutínská et al. 2021). This has been recently fostered with the genome sequencing of large collections of wild accessions sampled in different populations across Eurasia for two Brassicaceae plants, *A. thaliana* and *Cardamine hirsuta*, that diverged ∼32 Mya (1001 Genomes Consortium 2016; Baumgarten et al. 2023). They are both annual and autogamous plants with largely overlapping ecological niches (Hay et al. 2014), but show divergent trichome morphology, as *C. hirsuta*, like half of the Brassicaceae tribes, exclusively develops unbranched trichomes (Tutin et al. 1993; Huang et al. 2016). These resources are now opening the comparative genomics of natural intraspecific diversities through phenotypic genome-wide association studies (GWAS), which has already identified the *FRI/FLC* module as responsible of the convergent evolution of flowering time (Cartolano et al. 2015; Baumgarten et al. 2023). Furthermore, environmental genome-wide association studies (EGWAS) are beginning to reveal potential environmental factors that might drive such adaptations (Tabas-Madrid et al. 2018; Lasky et al. 2023).

Aiming to reveal the extent of convergent adaptation of trichome pattern in related species, in this study we have addressed the genomic mechanisms and environmental drivers underlying the natural diversity of *C. hirsuta*, as a relative of *A. thaliana*. To this end, we have developed and characterized a collection of 123 *C. hirsuta* accessions, but at a regional scale in the Iberian Peninsula, which contains two of the three major genetic lineages described in Europe (Baumgarten et al. 2023). We have previously analyzed a highly overlapping Iberian collection of *A. thaliana* embracing the largest European diversity (1001 Genomes Consortium 2016) across a wide altitudinal, climatic and ecological range (Castilla et al. 2020). Therefore, these regional resources provide an ideal scenario for comparative analyses of adaptive traits. The trichome pattern diversity of this *C. hirsuta* collection has been dissected by phenotypic and environmental GWAS, as well as by phenotype-environment regression analyses to address two main questions: (i) which are the genomic bases of leaf trichome pattern variation and its plasticity at different temperatures; (ii) which is the adaptive relevance of trichome pattern diversity. Thus, we identified several known genes and new loci, including an allelic series of *ChGL1* that accounts for the parallel evolution of trichome pattern in both species. Furthermore, we find that *C. hirsuta* displays opposite climatic associations than *A. thaliana*, supporting that this trait might be involved in adaptations to partly distinct environments in the two plants.

## Results

### Genetic, geographic, and environmental structure of Cardamine nucleotide diversity

**Figure 1.**
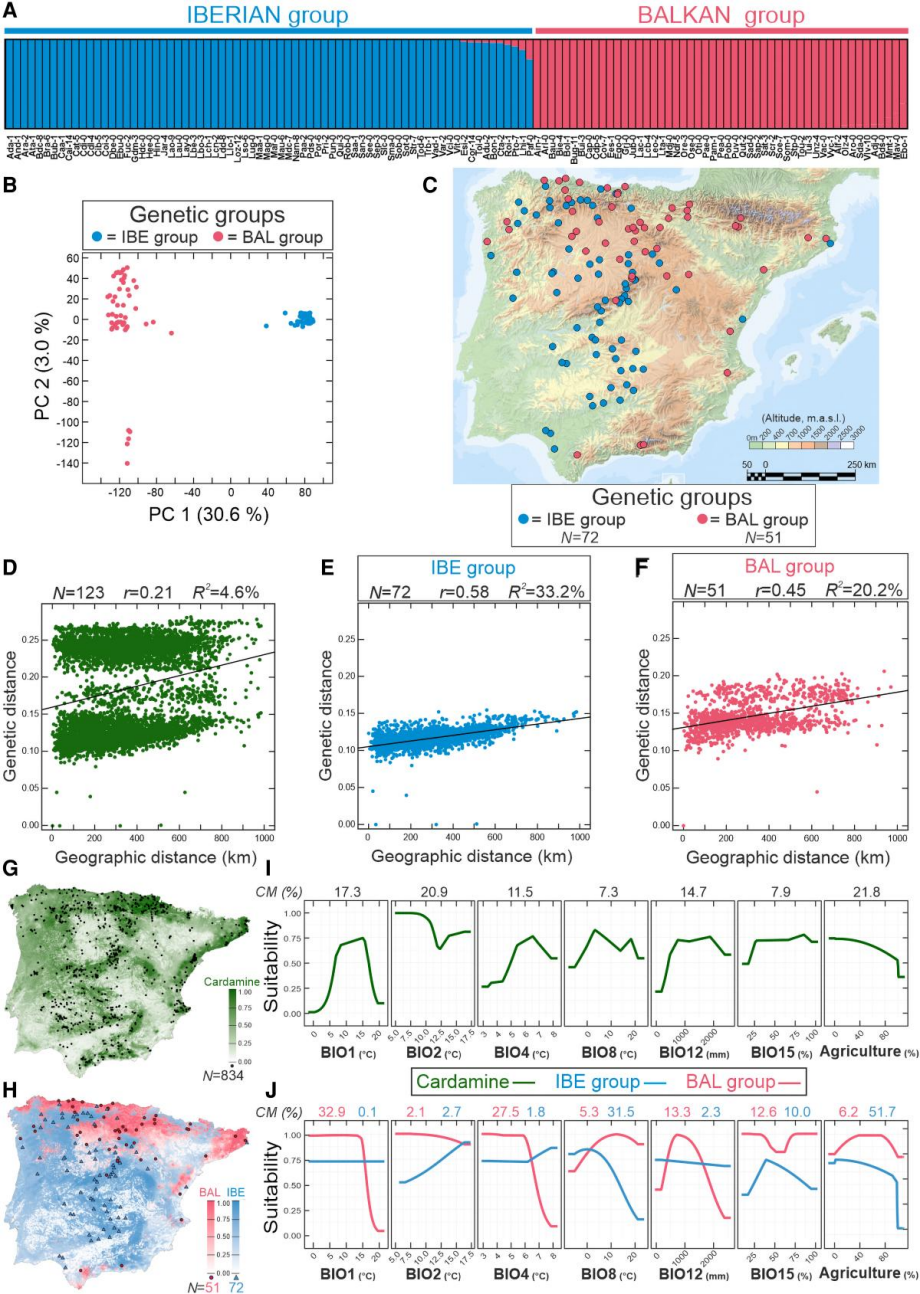
Genetic, geographic, and environmental structure of *C. hirsuta* populations. **A)** Genetic relationships among 123 accessions estimated for *K* = 2 ancestral genetic clusters with ADMIXTURE. Each individual is depicted as a vertical rectangle divided into segments representing the membership proportions estimated in the two ancestral clusters. Genotypes are arranged according to cluster membership proportions. **B)** Scatter plot displaying the PC analysis of the 123 genotypes. **C)** Geographic distribution of the IBE and BAL genetic groups detected by NJ, ADMIXTURE, and PC analyses. **D to F)** IBD for all 123 accessions (**D**), and for the IBE (**E**) and BAL (**F**) genetic groups. ADMIXTURE, PC, and IBD analyses were carried out using 343,364 nonsingleton SNPs with no missing data segregating in the accessions. **G, H)** Continuous potential distribution models of *C. hirsuta* quantifying habitat predicted suitability (adequacy of the species, or the genetic groups, to the environment where it occurs), in the Iberian Peninsula at the species level (**G**) and for the two genetic groups (**H**). *C. hirsuta* locations used to generate the distribution models are shown in the maps, whereas the number of samples are indicated in the corresponding suitability legends. **I, J**) Suitability response curves of the seven environmental variables used in *C. hirsuta* distribution models at the species level (**I**) and for the two genetic groups (**J**). The contribution of each environmental variable to the distribution models (CM%), measured as percentage drop in model fit when the variable is permuted, is shown on top of each panel. In all panels, IBE and BAL groups are blue and magenta colored, respectively, whereas the complete species is colored in green. BIO1: annual mean temperature; BIO2: mean temperature diurnal range; BIO4: temperature seasonality; BIO8: mean temperature of wettest quarter; BIO12: annual precipitation; BIO15: precipitation seasonality; %Agriculture: proportion of agriculture land per km^2^.

To characterize *C. hirsuta* genomic diversity at the regional scale of the Iberian Peninsula we analyzed 4.5 million single nucleotide polymorphism (SNPs) identified in the genome sequences of 123 accessions collected from different natural populations (Fig. 1; Supplementary Fig. S1; Supplementary Tables S1 and S2). To this end, we first determined the genetic relationships among these accessions using three complementary approaches: network clustering by neighbor-joining (NJ), the model-based clustering algorithm implemented in ADMIXTURE and the ordination method of principal components (PC). The three analyses found two highly diverse and differentiated genetic groups (*F_ST_* = 0.23 ± 0.28; Supplementary Table S3), which largely display nonoverlapping geographic distributions across this region (Fig. 1, A to C). Comparison of these clusters with those previously described in *C. hirsuta* at European scale (Baumgarten et al. 2023) showed that the 48 accessions shared in both studies precisely corresponded to the so-called Iberian (IBE) and Balkan (BAL) European groups, which appear mostly distributed in South-west and North-east Iberia, respectively. However, as shown by NJ and PC analyses, several accessions appeared weakly differentiated within BAL group (e.g. subgroup with low PC 2 values in Fig. 1B), although they did not show any geographic pattern (Supplementary Fig. S1). Therefore, mainly two highly differentiated genetic and geographic groups appear in Iberia, which parallels the regional structure of two major groups previously described in *A. thaliana* (Supplementary Fig. S2) (1001 Genomes Consortium 2016; Arteaga et al. 2021).

To better characterize the geographic distribution of genomic diversity we also analyzed *C. hirsuta* isolation-by-distance (IBD) patterns, which revealed a significant but moderate correlation between geographic and genetic distances at the species level (*r* = 0.21; *P* < 0.001) (Fig. 1D). However, as expected from the high genetic and geographic differentiation between IBE and BAL groups, these correlations were higher for each of the two genetic groups separately (Fig. 1, E and F). In particular, a stronger correlation was found for IBE (*r* = 0.58; *P* < 0.001; Fig. 1E) than BAL accessions (*r* = 0.45; *P* < 0.001; Fig. 1F) suggesting that IBE genetic lineage has a more ancient history in this region than BAL cluster.

We further dissected *C. hirsuta* geographic distribution and structure into environmental factors by estimating potential distribution models at species and genetic group levels (Fig. 1, G to J; Supplementary Table S4). Six bioclimatic and one landscape variables were included as environmental predictors of the current ecological distribution of *C. hirsuta* in the Iberian Peninsula (Fig. 1I; Supplementary Fig. S3). The potential distribution range of the species was mainly explained by the proportion of agricultural land and by three bioclimatic variables. Overall, *C. hirsuta* was more likely to occur in habitats with low percentage of agricultural land, moderate annual precipitation (800 to 1,800 mm; BIO12), moderate mean annual temperature (10 to 17 °C; BIO1), and low mean temperature diurnal range (<11 °C; BIO2) (Fig. 1I). However, the potential distribution of the two genetic clusters was explained by different environmental factors. The BAL cluster occurs mainly in habitats with low or moderate mean annual temperature (<15 °C), low temperature seasonality (<6 °C; BIO4), and moderate annual precipitation (600 to 1,200 mm) (Fig. 1, H to J). By contrast, IBE group showed low preference with respect to mean annual temperature or total precipitation. This group was mostly associated with low mean temperature of wettest quarter (<10 °C; BIO8) and absent in areas with high percentage of agricultural land (>80%) (Fig. 1, H to J). Thus, potential distribution models suggest that both genetic groups might be adapted to partly different environmental conditions.

### Ontogenic and genetic variation for leaf trichome pattern

**Figure 2.**
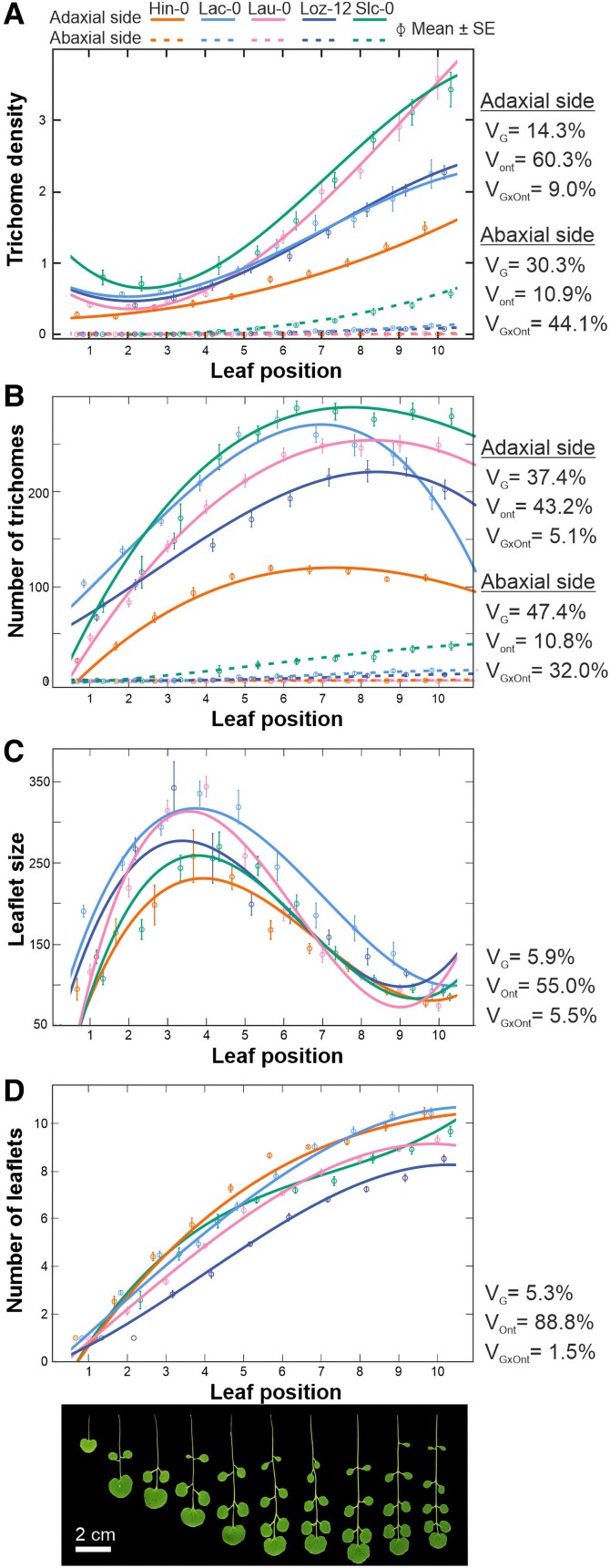
Natural variation for leaf trichome pattern across ontogeny. **A to D)** Leaf trichome density (**A**), leaf trichome number (**B**), terminal leaflet size (**C**), and leaflet number (**D**) in the first 10 rosette leaves of five *C. hirsuta* accessions. Each panel shows the mean ± standard error of each trait measured on each accession and fitted curves across the 10 leaves. Each accession is depicted with a different color according to legend, whereas continuous and dashed lines correspond to trichome traits for the adaxial and abaxial leaf surfaces, respectively. Phenotypic variances explained by the genotype (*V*_G_), the ontogeny (leaf position; *V*_Ont_) and the interaction between both factors (*V*_GxOnt_) are shown next to each panel. Representative leaves from the Slc-0 accession are shown below panel **D**, where images were digitally extracted for comparison.

As a first step to analyze the natural diversity of *C. hirsuta* for leaf trichome pattern, we selected five accessions across Iberia and studied the variation for trichome traits along ontogeny in both, adaxial and abaxial, leaf surfaces. Since *C. hirsuta* develops compound leaves that increase the number of leaflets across vegetative ontogeny (Cartolano et al. 2015), we focused on the terminal and largest leaflet. We quantified the number of leaflets, as well as the size (LS), the trichome number (TN), and the trichome density (TD) of the terminal leaflet in the first 10 leaves (Fig. 2). In the five accessions, the number of leaflets increased linearly along ontogeny, whereas terminal leaflet size increased only in the first four to five leaves, to decrease in the subsequent vegetative phase (Fig. 2, C and D). Therefore, most variation for these two leaf morphological traits occurred among leaves, and only 5% to 6% was explained by the genotypes (Supplementary Table S5).

Trichome traits also showed a differential behavior along vegetative development. In the leaf adaxial face, TN increased during the first five leaves of all accessions, but remained constant or decreased thereafter (Fig. 2B). On the contrary, TD was low and constant in leaves 1 to 5, but increased strongly thereafter (Fig. 2A). In contrast to the upper surface, the abaxial side of all accessions developed very few or no trichomes. Accordingly, trichome traits in the two leaf surfaces were not correlated since Lau-0 showed the highest density in the upper side but no trichomes in the adaxial face (Fig. 2, A and B). In addition, trichome traits in both sides showed substantial variation among accessions, genotypes accounting for 37% to 47% and 14% to 30% of the phenotypic variances for TN and TD, respectively (Fig. 2, A and B).

Overall, the behavior of leaf morphology and trichome traits distinguished two ontogenic stages in the vegetative development of *C. hirsuta*, which can be described as juvenile and adult phases (Fig. 2). The first five juvenile leaves are characterized by low leaflet number, increased size, and TN in the terminal leaflet, and a rather steady and low TD in the adaxial surface. By contrast, in subsequent leaves, the size of the terminal leaflet decreases, TN becomes steady and, consequently, the adaxial TD increases. From this ontogenic variation, trichome traits in the rest of this study were analyzed in the adaxial surface of leaves 6 to 7 because it maximized the genetic variation among accessions (Fig. 2, A and B).

### Natural diversity for leaf trichome pattern at different temperatures

**Figure 3.**
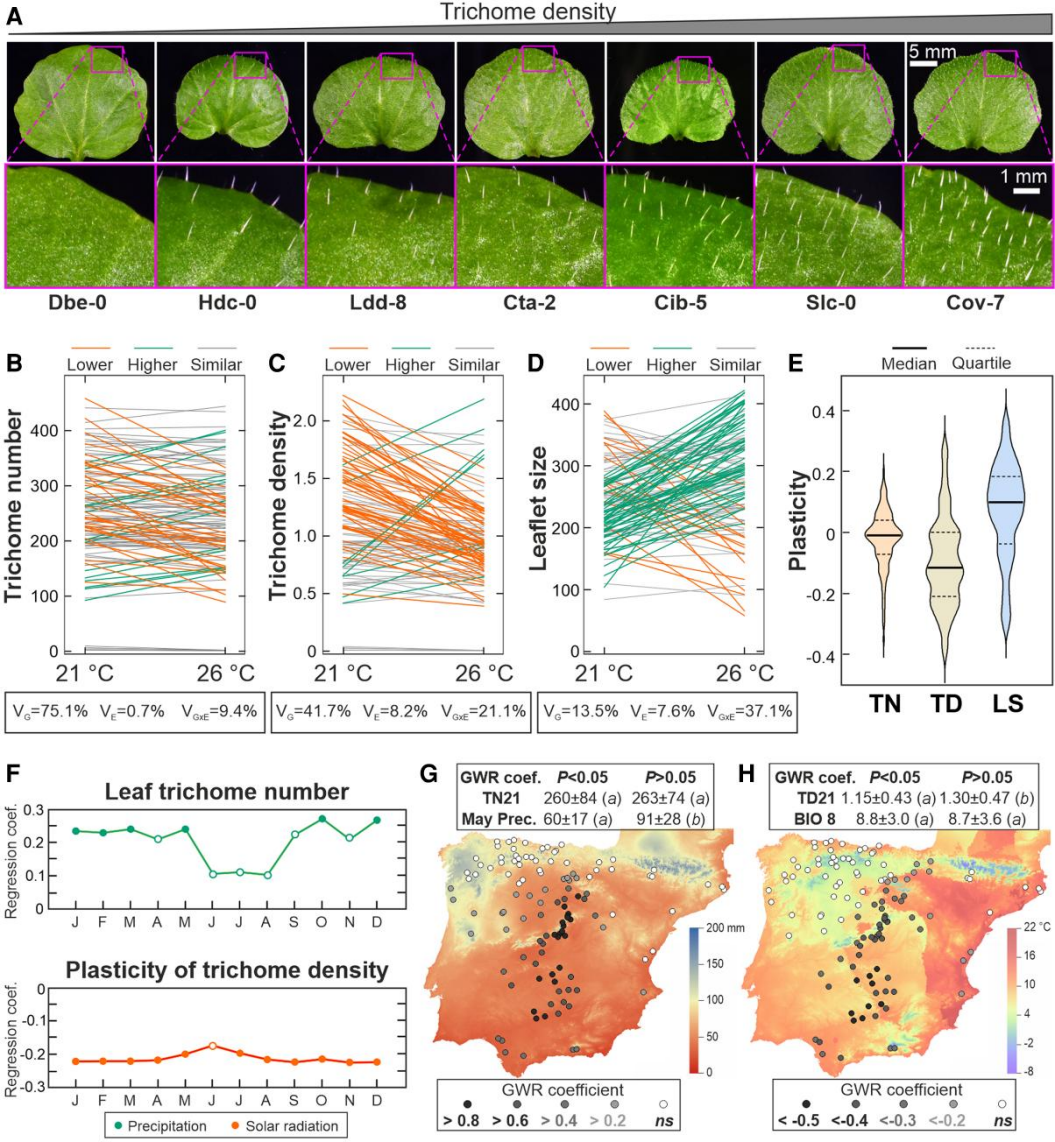
Natural variation and environmental associations for leaf trichome traits. **A)** Terminal leaflets of wild accessions with different trichome patterns arranged from glabrous to high trichome density. Close ups of insets are shown in lower panels. Leaf images were digitally extracted for comparison. **B to D)** Reaction norms of TN (**B**), TD (**C**), and LS (**D**) measured at 21 and 26 °C. As described in the legends, accessions are classified according to their similar or different phenotypes in both temperatures. Boxes in the lower part of each panel display the variance explained by the genotypes (*V*_G_), the environments (*V*_E_) or the interaction between both factors (*V*_GxE_). **E)** Violin graphs showing the variation for the plasticity of TN, TD, and LS to ambient temperature. **F)** Relationship between trichome traits and monthly precipitation (upper panel) or solar radiation (lower panel) along the year. Months in the abscissa are indicated with the first letter of the month. Filled and white circles depict significant (*P* < 0.05) and nonsignificant (*P* > 0.05) regressions, respectively. **G, H)** GWR analyses between May precipitation and trichome number at 21 °C (TN21) (**G**) or between mean temperature of wettest quarter (BIO8) and trichome density at 21 °C (TD21) (**H**). Panels show climatic maps including the GWR standard coefficients estimated at each location and depicted with different colors according to the legends. In the upper box of (**G**) and (**H**) panels, mean values ± standard deviation of trichome and climate variables are shown for locations with significant or nonsignificant GWR coefficients (indicated as *P* < 0.05 and *P* > 0.05, respectively). Differences between both types of locations for these variables were statistically tested by general linear models; the same or different letters indicate nonsignificant or significant differences (*P* < 0.05).

To determine *C. hirsuta* variation for leaf trichome traits at different growth ambient temperatures, we phenotyped the 123 Iberian accessions for trichome number (TN21 and TN26), trichome density (TD21, TD26), and terminal leaflet size (LS21, LS26) at 21 and 26 °C. Five accessions developed no trichome indicating that glabrousness occurs in some natural populations of *C. hirsuta*, and at a similar low frequency of 0.04 than in *A. thaliana* in this region (Arteaga et al. 2022). The remaining accessions displayed a 4-fold variation between the extreme phenotypes (Fig. 3, A to D). All traits showed substantial genetic variation among accessions and moderate to high heritabilities at both temperatures, TN traits displaying the highest values (Table 1; Supplementary Table S5). Comparisons of traits between both temperatures showed that, on average, accessions have lower number of trichomes but larger leaflet size at 26 than 21 °C, which leads to a larger reduction of TD at high temperature (Fig. 3, B to D). However, analysis of the individual reaction norms revealed significant genotype by environment (temperature) interactions (Fig. 3, B to D) because we found accessions that significantly increased or reduced the various traits at high temperature (*P* < 0.01). In agreement with the average values, high temperature increased leaflet size and reduced TN in 42% and 26% of the samples, respectively (Fig. 3, B to D). By contrast, only 12% of these accessions had significantly smaller leaflet size, or higher TN at 26 than 21 °C. Accordingly, high temperature reduced TD in 46% of the accessions, whereas it increased this trait only in 7% of the genotypes (Fig. 3C). This genetic variation led to moderate correlations among the various traits measured at similar or different temperatures (0.5 < *r* < 0.7; *P* < 0.01), and only TN21 and TD21 or TN26 showed high Pearson coefficients (*r* > 0.7; *P* < 0.001; Supplementary Table S6; Supplementary Fig. S4). These results indicate that, despite the direct relationship between trichome number, density and leaflet size, partly independent genetic bases underlie most of the traits.

**Table 1.** Summary of GWAS of trichome pattern traits

Trait	Mean ± Sd	*h^2^_b_*	*h^2^_kinship_*	*h^2^_10 loci_*	Number of SNPs	Number of genes	Trichome pattern candidate genes^a^
TN21	262 ± 80	83.8	48.8	86.5	198	115	*ChGL1*, *ChSAD2*, *ChTOE1*, *ChBHLH87*^b^
TN26	249 ± 77	86.4	39.2	67.2	43	35	*ChGL1*, *ChTOE1*, *ChBHLH87*^b^, *ChBHLH96*^b^
TD21	1.22 ± 0.45	66.9	36.6	79.0	197	64	*ChGL1*, *ChETC3, ChMYB15*^b^*, ChURO*^b^
TD26	0.99 ± 0.33	72.2	24.4	75.2	182	105	*ChMYB23*, *ChNTL8*, *ChADL1*
PTN	−0.023 ± 0.097	…	13.3	75.2	1132	161	*ChNTL8*
PTD	−0.100 ± 0.147	…	29.8	60.9	116	70	*ChETC3*, *ChMYB23*, *ChLEC2*, *ChTOE2*

For each trait is shown: the mean ± Sd; the broad sense heritability (*h*^2^*_b_*); the variance explained by the kinship matrix (*h*^2^*_kinship_*) or the 10 most strongly associated loci (*h*^2^*_10 loci_*); the number of significant SNPs; the number of associated genes; and a list of candidate genes related with trichome pattern regulation. ^a^*C. hirsuta* orthologues or homologues of *A. thaliana* genes known to affect trichome pattern. ^b^*C. hirsuta* homologues of *A. thaliana* genes known to affect trichome pattern.

To better understand the natural variation for the response of these traits to temperature at the species level, we also estimated the plasticity of TN, TD, and LS (PTN, PTD, and PLS) to temperature (Fig. 3E). Overall, positive (increase at 26 °C) and negative (decrease at 26 °C) values were estimated, respectively, for PLS and PTN, which led to larger negative PTD values. Therefore, in *C. hirsuta*, high ambient temperature tends to increase leaflet size, and to reduce TN and TD, although natural populations show broad genetic variation for all traits and their responses to temperature.

Furthermore, comparisons of the two *C. hirsuta* genetic groups identified in Iberia did not reveal major differences for most traits, including TN and TD at both temperatures (*P* > 0.01; Supplementary Table S5). In agreement, traits showed low quantitative genetic differentiation between IBE and BAL groups (*Q_ST_ =* 0% to 11.5%). Both groups only differed significantly for LS26, PLS and PTD, with IBE lineage showing larger LS at 26 °C (IBE = 296 ± 62; BAL = 256 ± 99) and stronger plasticity of LS (IBE = 0.12 ± 0.12; BAL = 0.01± 0.17) and TD (IBE = −0.14 ± 0.12; BAL = −0.04 ± 0.16) to temperature. Thus, most Iberian genetic variation for leaf trichome traits appeared distributed across the geographic range of each genetic group.

### Geographic and climatic distribution of leaf trichome pattern diversity

We further assessed the adaptive relevance of the genetic variation for trichome traits, by analyzing geographic autocorrelations and associations with the climatic diversity across this region. No significant Moran's *I* autocorrelations were detected, thus indicating that trichome traits show no overall spatial structure. In addition, we analyzed the relationship between trichome traits and annual and monthly temperatures, precipitations and solar radiations by applying spatial autoregressive models, which take into account the spatial structure of both dependent and independent variables. Trichome traits measured at 21 °C showed significant correlations with precipitation parameters, whereas no association was detected for traits at 26 °C (Supplementary Table S7). In particular, TN21 correlated positively with winter and spring precipitations, as well as with total annual rain (Fig. 3F). In addition, TD21 and PTN appeared weakly or marginally associated (0.03 < *P* < 0.06) with the mean temperature of the wettest quarter (BIO8), such that locations with low temperature correspond to high TD21 and strong negative PTN responses to temperature (Supplementary Table S7). Furthermore, PTD was negatively correlated with solar radiation throughout the year (Fig. 3F) further supporting that climate is a relevant driver of the *C. hirsuta* diversity for trichome traits.

To better understand the relationships between trichome traits and the most significant climatic variables (May precipitation and BIO8) we carried out geographically weighted regressions (GWR) at lower spatial scale. GWR estimates a local coefficient for each of the population locations by taking into account only a fraction (10% to 15%) of the samples corresponding to neighboring locations selected at an optimized geographic distance. These analyses detected stronger relationships for TN21, TD21, and PTN traits, but coefficients varied greatly across geography (Fig. 3, G and H; Supplementary Fig. S5). TN21 and TD21 showed maximum positive regressions with May precipitation in central Iberia, which correspond to locations with significantly lower precipitation (*P* < 0.009; Supplementary Table S5). However, trichome traits did not differ between locations with significant and nonsignificant coefficients (Fig. 3G; Supplementary Fig. S5). By contrast, TN21 and TD21 showed significant negative regressions with BIO8 in southern locations characterized by lower trichome trait values (Fig. 3H; Supplementary Fig. S5), but not differing from other locations for the climatic variable (*P* > 0.48; Supplementary Table S5). In addition, regressions between PTN and BIO8 showed a different geographic pattern, and locations with significant regressions displayed higher BIO8 temperature (Supplementary Fig. S5; Supplementary Table S5). Further analyses of GWR coefficients also detected significant differences between IBE and BAL genetic groups for all pairs of trait/climatic factors tested (*P* < 0.006; Supplementary Table S5). Accessions belonging to IBE group displayed stronger climatic associations for TD21 and TN21, whereas BAL group had stronger regressions for PTN. Therefore, the relationships between leaf trichome traits and climate depend on multiple factors that vary across geography.

### GWAS of leaf trichome pattern and climatic variables

**Figure 4.**
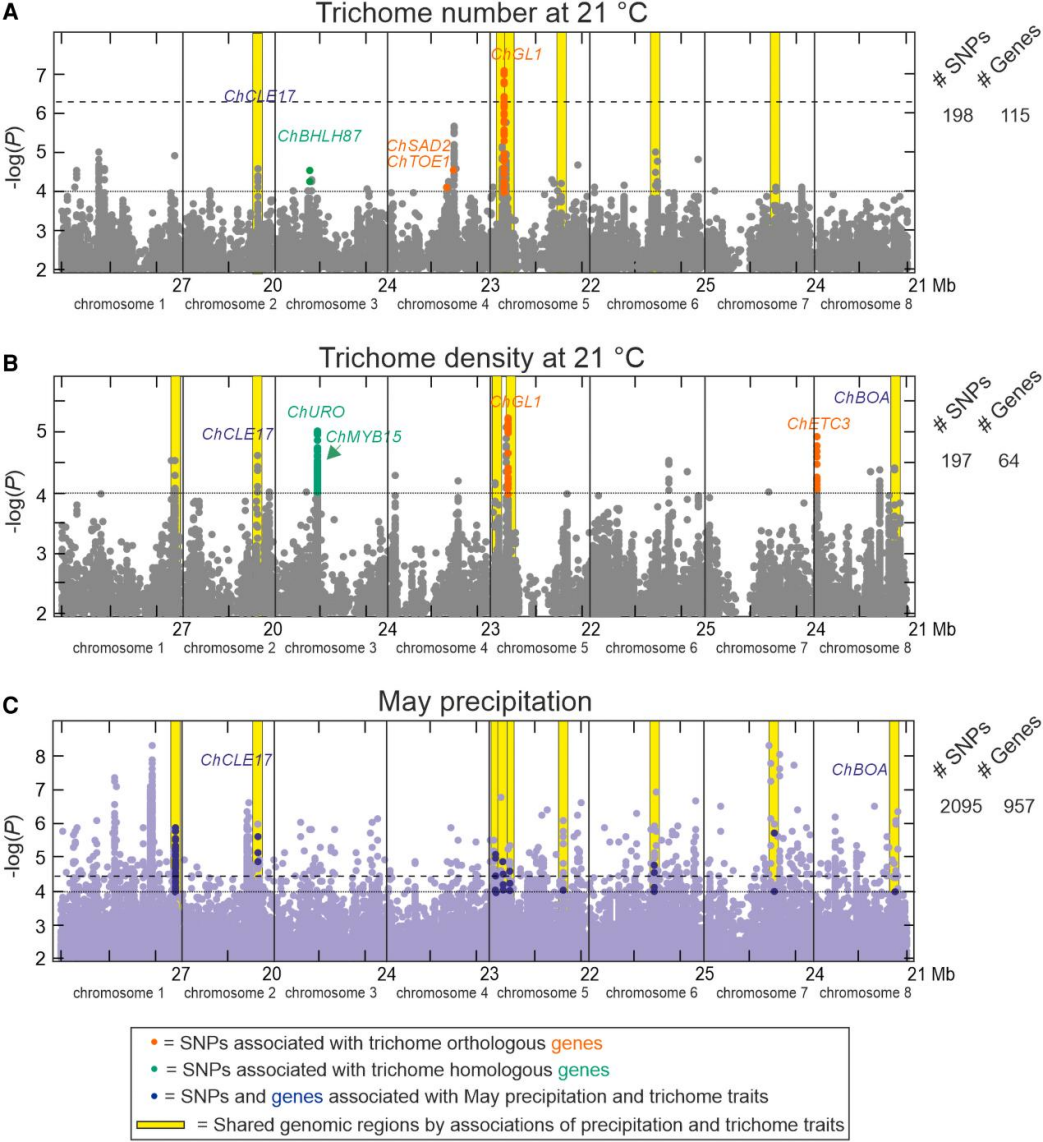
Phenotypic and environmental genome-wide association analyses. **A to C)** Manhattan plots for leaf TN (**A**) and TD (**B**) measured at 21 °C, as well as for May precipitation (**C**), across the eight *C. hirsuta* chromosomes. Horizontal black dotted and dashed lines indicate significance thresholds of −log(*P*) = 4 and FDR = 0.1 after Benjamini–Hochberg correction for multiple tests, respectively. Orange and bluish green colored dots match SNPs with −log(*P*) > 4 that are located on *C. hirsuta* orthologues or homologues, respectively, of *A. thaliana* genes known to affect trichome development; the names of these genes are included in each panel. Blue colored dots depict SNPs with −log(*P*) > 4 that are located on *C. hirsuta* genes associated with both, trichome traits and May precipitation; the names of the two top genes associated with trichome traits are included. Yellow color strips depict significant genomic regions associated with trichome and precipitation variables.

To identify potential mechanisms that might explain *C. hirsuta* trichome diversity, we carried out GWAS for TN and TD and their plasticities to temperature using 3.3 million SNPs segregating in the 118 nonglabrous accessions. Trichome traits showed low to moderate correlations with the genomic background, as measured by the phenotypic variance explained by a kinship matrix (Table 1). Despite these low correlations, only one genomic region located on chromosome 5 was detected at a high significance threshold of −log(*P*) > 6.3 (corresponding to FDR = 0.1 after Benjamini–Hochberg correction for multiple tests) for TN21 (Fig. 4; Supplementary Fig. S6). At a lower statistical significance of −log(*P*) > 4, a total of 43 to 1,132 SNPs located in 35 to 161 genes were associated with the different quantitative traits (Table 1). As expected from the moderate correlations found between traits at 21 and 26 °C, only 36% and 10% of the associated genes were shared at both temperatures for TN and TD, respectively (Supplementary Fig. S7). Further comparisons of the 10 top genomic regions detected for each trait identified five regions shared among traits (Supplementary Table S8). In particular, the most significant region was common to TN21, TD21, and TN26, with the strongest SNP associations locating on gene *CARHR149430*, which is the orthologue of *A. thaliana GL1* (Fig. 4; Supplementary Fig. S6). In addition, the orthologue of *A. thaliana ETC3* was located in the third most strongly associated region with TD21. Moreover, genes *ChMYB15*, *ChBHLH87*, *ChBHLH96*, and *ChURO*, encoding transcription factors of major families affecting trichome pattern in multiple species, appeared among the top genes associated with TN and TD at different temperatures (Fig. 4; Supplementary Fig. S6; Table 1).

To find other relevant candidate genes with effect on trichome traits, we also analyzed the genomic regions around 149 *C. hirsuta* genes that are orthologues of *A. thaliana* genes known to be involved in trichome development (Supplementary Table S9). In addition to *ChGL1* and *ChETC3*, we detected seven genes including the *GL1* homologue *ChMYB23* and the NAC transcription factor *ChNTL8*, both affecting trichome pattern in *A. thaliana* (Table 1). SNPs in *ChGL1*, *ChETC3* and several other candidate genes (*ChMYB15*, *ChBHLH96*, *ChURO*, *ChMYB23*, and *ChNTL8*) appeared associated with TN or TD traits only at 21 or 26 °C, and they showed significant interaction with temperature (Supplementary Table S8). Furthermore, all regions detected for PTN and PTD also interacted significantly with temperature (*P* < 0.01), supporting that these genes might contribute to the genotype by temperature interactions described for trichomes traits.

Finally, to test if these genomic regions might also contribute to adaptation to the main climatic variables correlating with trichome traits (May precipitation, BIO8 and BIO12) we carried out EGWAS. These analyses identified 181 to 957 genes associated with those climatic variables (Fig. 4C). Comparisons of the genes detected by GWAS of the four TN and TD traits and EGWAS of the three climatic variables, identified a total of 17 genes located in 11 genomic regions significantly associated with both sets of variables (Fig. 4; Supplementary Table S8). All of them correlated with precipitation variables, and only three were also associated with BIO8. Two of these genomic regions flank *ChGL1* gene at 100 to 400 kb distances, although *ChGL1* showed no significant climatic association (−log(*P*) = 2.9). By contrast, two of the remaining 10 top genes associated with TN21 and TD21, *ChCLE17* and *ChBOA*, were also associated with precipitation (4 < −log(*P*) < 5.6) (Fig. 4), although a low number of SNPs showed significant associations. Despite this limitation, in agreement with the trichome/climate correlations, in both genes, alleles increasing trichome traits also associated with higher precipitation, which suggests that they might be involved in climatic adaptation through modifications of trichome pattern (Supplementary Fig. S8).

### Functional characterization of a *ChGL1* allelic series affecting leaf trichome pattern

**Figure 5.**
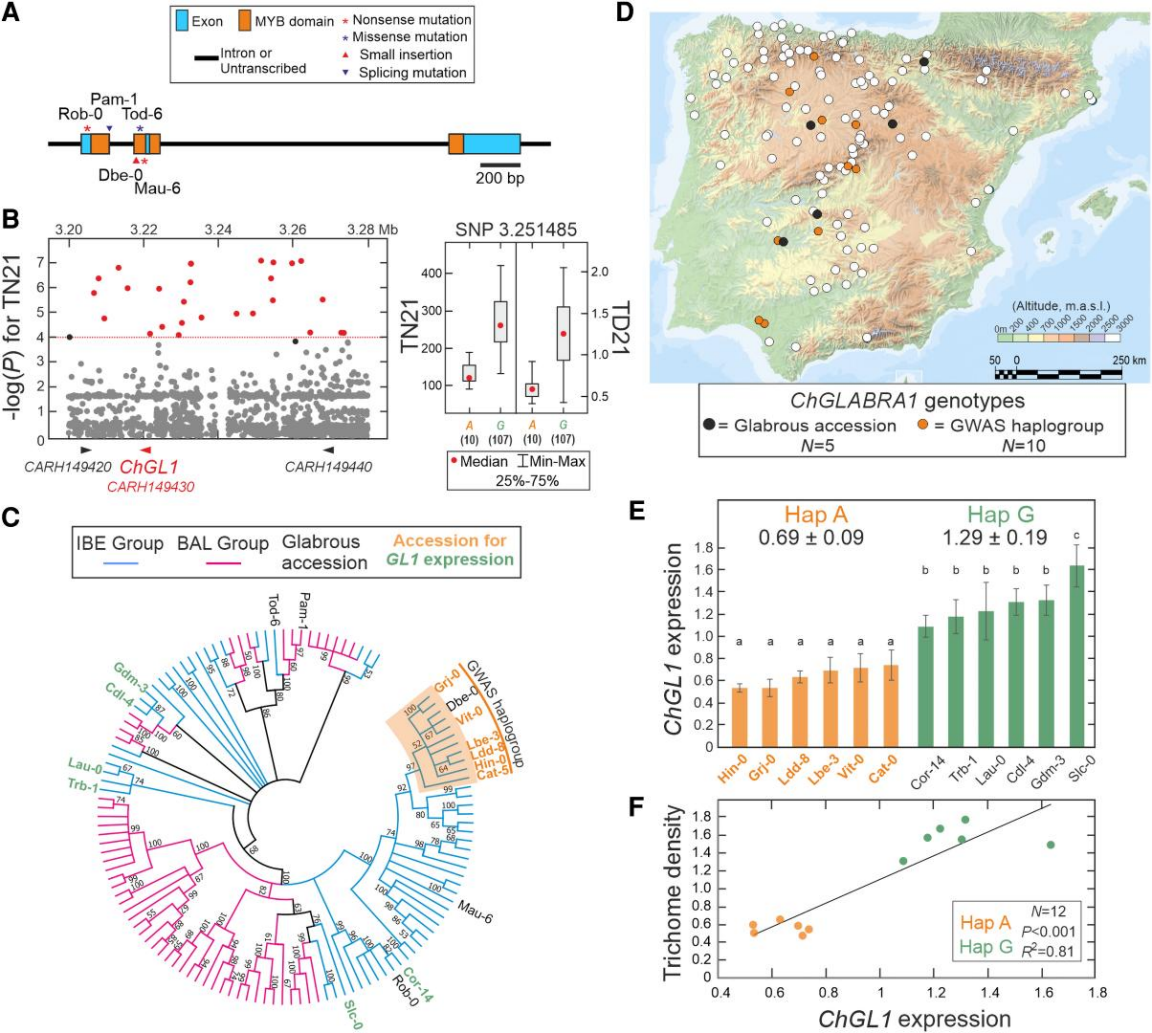
Functional analyses of *ChGL1*. **A)***ChGL1* loss-of-function mutations causing *C. hirsuta* glabrousness in the Iberian Peninsula. **B)** Zoom of Manhattan plot around *ChGL1* and effects of the most significantly associated SNP on trichome number (TN21) and density (TD21) measured at 21 °C. Red colored dots in Manhattan plot match SNPs showing associations above the −log(*P*) > 4 threshold depicted as dotted line. **C)** Topology of NJ tree displaying *ChGL1* genetic relationships among accessions. Branches corresponding to partitions reproduced in <50% bootstrap replicates are collapsed, whereas branches corresponding to IBE or BAL groups are colored in blue and magenta, respectively. Glabrous accessions or those selected for gene expression analysis are indicated in NJ tree. **D)** Geographic distribution of Iberian accessions belonging to the *ChGL1* haplogroup detected by GWAS (named as GWAS haplogroup), or carrying glabrous alleles. The number of accessions in each class is indicated in the legend. **E)***ChGL1* expression analysis in 12 accessions differing in *ChGL1* SNPs (Hap A and G) differentiating GWAS haplogroup. Bars show the mean ± Se of three biological replicates per accession. Relative gene expression differences among genotypes were statistically tested by mixed linear models, the same or different letters indicating nonsignificant or significant differences in Tukey's test (*P* < 0.05). **F)** Linear regression between TD21 and *ChGL1* relative expression.

Since *ChGL1* was the most relevant candidate gene associated with different trichome traits, we characterized its natural diversity. Sequencing the *ChGL1* coding region in the five glabrous accessions identified one small insertion and one splicing mutation predicted to generate truncated proteins, two nonsense mutations, and one missense mutation in a highly conserved gene region (Fig. 5A). Most of these mutations affected the conserved MYB domains of the protein, as previously described for glabrous accessions of *A. thaliana* (Supplementary Fig. S9). Genetic analysis of an F_2_ population derived from a glabrous (Tod-6) and a hairy (Slc-0) accession showed monogenic segregation, as well as absolute co-segregation between glabrousness and the *ChGL1*-Tod-6 missense mutation (Supplementary Fig. S10). This indicates that such qualitative trait is caused by five independent complete loss-of-function (null) mutations in *ChGL1* gene, and that *ChGL1* functions as an activator of trichome development in *C. hirsuta*. Analysis of *ChGL1* genomic region in the remaining 118 hairy accessions used for GWAS showed that all 23 SNPs most significantly associated with trichome traits were located in the 63 kb of promoter and 3′ regulatory regions flanking *ChGL1* gene (Fig. 5B). This indicates that the linkage disequilibrium in *ChGL1* region is stronger than the average values described for *C. hirsuta* Iberian groups, which extends up to 20 kb (Baumgarten et al. 2023). Phylogenetic analysis of the nucleotide diversity of this region revealed a general *ChGL1* differentiation of IBE and BAL genetic groups (Fig. 5C). In addition, it identified a haplogroup of 10 hairy accessions of the IBE lineage that were differentiated by those 23 GWAS SNPs, but showed no missense mutation in *ChGL1* coding sequence. The accessions belonging to this GWAS haplogroup had lower TN and TD, with the GWAS SNPs accounting for 19% to 27% of the phenotypic variance for these traits (*P* < 0.0001; Fig. 5B). These results suggest that one or several *cis*-regulatory mutations shared by these 10 accessions and in linkage disequilibrium with the GWAS SNPs reduce the function of this *ChGL1* haplogroup. We further explored this hypothesis by analyzing *ChGL1* expression in a set of 12 IBE accessions, finding that, on average, expression was half in GWAS haplogroup than in other accessions (Fig. 5E; Supplementary Table S5). Moreover, *ChGL1* expression strongly correlated with TD, and accounted for 81% of the variation in this sample (Fig. 5F). Hence, accessions of this haplogroup carry a partial loss-of-function allele of *ChGL1* that is caused by *cis*-regulatory mutations. Analyses of the geographic and climatic distribution of both, complete and partial, loss-of-function alleles of *ChGL1* showed no significant association (Figs. 4C and 5D). Therefore, other environmental factors that do not correlate with the regional climate, such as specific local biotic agents, or random genetic drift, likely contribute to maintain *ChGL1* diversity across Iberia.

## Discussion

### The Iberian Peninsula as a highly diverse region for comparative analyses of Cardamine and Arabidopsis

To understand the mechanisms underlying plant adaptation to different environments, we have carried out a regional study of the natural diversity for nucleotide polymorphisms and trichome pattern traits in *C. hirsuta* in the Iberian Peninsula. Several results from the comparison of regional and global patterns of genetic diversity, differentiation, and structure suggest a complex demographic and adaptive history of *C. hirsuta* in this region, including multiple colonization events from different European refugia. First, the presence of two highly diverse and differentiated genetic groups, IBE and BAL, previously estimated to have split 319 kya (Baumgarten et al. 2023), make this region a hot spot for *C. hirsuta* diversity. Second, the higher frequency of both genetic groups in Iberian and Balkan Peninsulas, respectively (Baumgarten et al. 2023), suggests the occurrence of old refugia for each group in those regions. Third, the similar strong genetic differentiation among IBE and BAL lineages at Iberian and European scales (*F_ST_* = 0.23 ± 0.28 and 0.25 ± 0.08, respectively) indicates that both groups have been rather isolated in this region despite their geographic proximity, thus suggesting a recent colonization of Iberia by BAL group spread from Eastern Europe. This is also supported by the weaker IBD pattern detected for BAL than IBE group, in agreement with a more recent expansion of BAL lineage in this region. Accordingly, we hypothesize that an ancient colonization by IBE lineage before the last glaciations, and a more recent postglacial colonization by BAL group, account for the high diversity and strong geographic structure of *C. hirsuta* in Iberia. Interestingly, a parallel demographic history has been proposed for *A. thaliana* in Europe, with an old genetic lineage refuged mainly in Iberia and so-called Relict, and a postglacial East to West colonization of a Nonrelict group spreading from the Balkan and Caucasus area about 10 to 45 kya (Supplementary Fig. S2) (Lee et al. 2017; Fulgione and Hancock 2018). Both highly differentiated *A. thaliana* lineages have been traced back to Africa (Durvasula et al. 2017). However, the geographic origin of *C. hirsuta* and its African history remain unknown, awaiting future studies beyond Europe.

In addition to complex demographical processes (e.g. expansion and bottle neck cycles) caused by large environmental changes (e.g. multiple glacial and interglacial periods), evolutionary adaptations to past and current environmental conditions have also contributed to shape the geographic and genetic differentiation of IBE and BAL lineages of *C. hirsuta* in Iberia. This is supported by the potential distribution models explained by different ecological and climatic factors. In particular, the higher occurrence of BAL group in highly agricultural landscapes suggests that the spreading of this lineage across Europe might be more associated to the expansion of human agriculture, as proposed for the Nonrelict lineage of *A. thaliana* (François et al. 2008; Lee et al. 2017). Moreover, the stronger suitability of habitats with high mean annual temperature or low precipitation for IBE than BAL group, supports that both groups might also be adapted to different climatic conditions. Interestingly, the substantial diversity quantified for trichome pattern traits in both groups likely contributes to this adaptation, although in a lineage specific manner because traits correlated differentially with climatic factors in IBE and BAL groups. GWR results further support that adaptation of trichome traits varies across geography, in agreement with *A. thaliana* observations for trichome pattern and other adaptive traits (Tabas-Madrid et al. 2018; Arteaga et al. 2022). Such spatial heterogeneity indicates that additional factors interact with the genetic background to determine the relevance of trichome traits on adaptation, including: (i) the particular range of variation of environmental factors, as observed for the associations with precipitation; and (ii) specific local environmental components, as suggested by the regressions with BIO8 temperature. Therefore, the similarity between the evolutionary processes found to shape the natural diversity of *C. hirsuta* and *A. thaliana* at global and regional scales supports a long-shared demographic and adaptive history of both annual plants across Europe.

### Different environmental drivers shape the leaf trichome diversity of Cardamine and Arabidopsis

Despite the similar demographic and adaptive histories of *C. hirsuta* and *A. thaliana*, the ecological function of trichome traits seems largely distinct in both plants, as indicated by several results. On one hand, the analyses of leaf morphological and trichome traits along vegetative development show substantial but differential ontogenic variation in both species. In agreement with previous studies (Cartolano et al. 2015; Baumgarten et al. 2023), we found that *C. hirsuta* heteroblastic changes, such as the increase in leaflet number and, specially, the size reduction of terminal leaflets, largely differentiate juvenile and adult vegetative phases. Additionally, we show that the quantitative variation of TN and TD in the adaxial face also distinguishes both vegetative periods. By contrast, in *A. thaliana*, the transition from juvenile to adult leaves has been mainly characterized by the development of trichomes in abaxial leaf surfaces (Walker and Marks 2000; Wang et al. 2019). However, such heteroblastic changes in the trichome pattern of the abaxial face do not reliably mark this vegetative transition in *C. hirsuta* due to its large variation among accessions. Nevertheless, abaxial trichomes have been mainly studied in *A. thaliana* laboratory strains, Col and L*er*, leaving open the question of how this trait behaves in other natural accessions.

On the other hand, the natural diversity of trichome traits appears as involved in adaptation to different climatic factors in *C. hirsuta* and in *A. thaliana*. In agreement with the leaf plasticity described for *A. thaliana* and other plants in relation to moderate ambient temperatures (16 to 25 °C) (Lippmann et al. 2019), the terminal leaflet size of *C. hirsuta* increases at 26 °C compared to 21 °C. In addition, the plasticity of trichome pattern traits suggests that, in *C. hirsuta*, trichomes are involved in adaptation to low temperatures because an overall higher TD is found at low than high temperatures. This idea is further supported by the climatic distributions of trichome traits, since a nonrandom arrangement (significant association) of the trichome diversity in relation to climatic variables might reflect the effect of such environmental variables as, either direct or indirect, selective forces on the trait. In particular, high TN and TD appeared associated with high precipitation and low temperature in spring season. Moreover, high plasticity of trichome density in relation to temperature is associated with locations of low solar radiation. By contrast, a previous *A. thaliana* study in the same region and locations has shown that trichome traits are correlated with similar climatic variables, but in opposite direction because high trichome density appears in populations with low precipitation, high temperature, and high radiation (Arteaga et al. 2022). Analogous climatic associations in numerous plants with branched trichomes, such as *A. kamchatica*, *Mimulus guttatus* or *Quercus* sp have suggested that trichomes may protect against abiotic stresses caused by water loss, drought, or excess UV radiation, as well as from biotic damages produced by herbivore insects favored by those climatic conditions (Steets et al. 2010; Kooyers et al. 2015; Mediavilla et al. 2019). On the contrary, as described here for *C. hirsuta*, high trichome density has been also correlated with low temperature or high precipitation in several plants with unbranched trichomes, such as maize, *Antirrhinum* and soybean, suggesting a protective role of trichomes against low temperature (Hufford et al. 2013; Tan et al. 2020) or reduced tolerance to drought stress (Liu et al. 2020). In addition, trichomes have been shown to facilitate growth of pathogenic and beneficial mycorrhizal fungi (Calo et al. 2006; Waller et al. 2018) and to affect the richness of the leaf bacterial community (Horton et al. 2014). Thus, *C. hirsuta* trichome pattern traits might also be involved in adaptation to beneficial or detrimental fungi and bacteria encouraged by high and low precipitations, respectively. Nevertheless, we cannot exclude that other environmental factors correlating with climatic variables might drive the differential evolution of trichome pattern in these species.

As illustrated with *C. hirsuta* and *A. thaliana* comparison, although trichomes have been involved in many adaptations, their ecological functions strongly depend on the plant species. The distinct environmental associations of unbranched and branched trichomes described for these and other plants suggest that not only trichome pattern, but also trichome morphology, provide specific mechanisms for plant adaptation to different environments. However, additional comparative studies are needed to further disentangle the complex interactions between abiotic and biotic environmental factors and genetic drift in shaping the diversity for trichome traits.

### Conserved genetic mechanisms account for the trichome diversity of Cardamine and Arabidopsis

The genomic and genetic dissection of trichome pattern traits has identified several *C. hirsuta* candidate genes whose orthologues also contribute to the natural variation in *A. thaliana* and other relatives. Accordingly, the trichome pattern diversity of Brassicaceae plants shows parallel genetic evolution, understood as the convergent evolution of similar phenotypes in different species caused by independent mutations in similar (orthologous or paralogous) genes (Stern 2013; Bohutínská et al. 2021). In particular, we identified a series of functional alleles with complete or partial loss-of-functions of *ChGL1* that account for the qualitative and quantitative variation of trichome traits. Five independent null mutations of *ChGL1* appear as responsible of the glabrous phenotype of several Iberian populations, which indicates repeated evolution of this qualitative trait in *C. hirsuta*. Similar results have been previously described for the orthologous *GL1* gene of *A. thaliana*, *A. lyrata*, *A. halleri* and several crop plants (Hauser et al. 2001; Kivimäki et al. 2007; Li et al. 2013; Xuan et al. 2020). This demonstrates a wide conservation of *GL1* function as promoter of trichome development in Brassicaceae tribes with unbranched and branched trichomes. In addition, characterization of the *ChGL1* haplogroup identified by GWAS suggests that other *cis*-regulatory mutations quantitatively fine tune *ChGL1* function and trichome pattern. A comparable allelic series of *GL1* hypo- and hypermorphic alleles has been described as contributing to the trichome pattern diversity of *A. thaliana*, although the latter alleles are caused by protein structure mutations (Supplementary Fig. S9) (Bloomer et al. 2012; Arteaga et al. 2021, 2022). Therefore, *GL1* emerges as a major gene accounting for the parallel evolution of trichome pattern variation in Brassicaceae.

GWAS also identified *ChETC3*, which shows strong homology (72% to 89% protein similarity) with *A. thaliana ETC2* and *TCL1* genes encoding R3 MYB transcription factors that repress trichome development (Wang et al. 2007; Wester et al. 2009). Both, *ETC2* and *TCL1*, have been shown to account for *A. thaliana* natural diversity of leaf trichome pattern (Hilscher et al. 2009; Arteaga et al. 2022), but they lack orthologues in *C. hirsuta* (this study; Walden and Schranz 2023). This suggests that, in *C. hirsuta*, the homologous gene *ChETC3* has paralleled the evolution of *A. thaliana ETC2* and *TCL1*, causing intraspecific diversity for leaf trichome pattern. In addition, several candidate genes encoding transcription factors known to regulate trichome development in numerous plants (Pattanaik et al. 2014; Fambrini and Pugliesi 2019, Han et al. 2022) might affect *C. hirsuta* trichome pattern and plasticity to temperature because they were only detected at 21 (*ChETC3*, *ChMYB15*, and *ChURO*), or 26 °C (*ChBHLH96*, *ChMYB23*, and *ChNTL8*). However, the lack of climatic associations for all these genes suggests that they might be involved in adaptation to more complex environmental conditions. By contrast, *ChCLE17* and *ChBOA*, regulating cell fate and signaling but not affecting trichome development in *A. thaliana* (Dai et al. 2011; Dao et al. 2022), were associated with both, trichome and environmental, variables. These genes then appear as potential candidates that might contribute to climatic adaptation through trichome pattern modifications.

Our comparative genetics of *C. hirsuta* and *A. thaliana* has identified several mechanisms accounting for the parallel evolution of trichome pattern in Brassicaceae. Similar parallel evolution has been also described for stem trichome pattern in various hairy *Antirrhinum* species adapted to alpine environments, although this appears as mediated by the *H* gene encoding an epidermis specific glutaredoxin (Tan et al. 2020). By contrast, previous Brassicaceae studies have suggested a *GL1* function in local adaptation to herbivory, which might involve heterogeneous selection in fluctuating environments and a fitness cost of trichome production (Mauricio and Rausher 1997; Kivimäki et al. 2007; Steets et al. 2010; Sato and Hudoh 2017; Sato et al. 2019; Xuan et al. 2020). However, we cannot discard that Brassicaceae diversity for trichome density is the result of a balance between positive selection on hairy phenotypes and genetic drift on *GL1* loss-of-functions. In conclusion, these molecular and environmental differences indicate that distinct evolutionary constrains likely drive the parallel evolution of trichome pattern in different eudicot families. Future comparative studies will elucidate the precise ecological and evolutionary mechanisms maintaining the intraspecific diversity for trichome patterns and genes, as well as the prevalence of such convergent adaptations in angiosperm plants.

## Materials and methods

### Plant material and environmental data

In this study we generated a *C. hirsuta* regional collection of 123 genetically distinct wild accessions from the Iberian Peninsula sampled from different georeferenced local populations (Supplementary Table S1). Together they span a region of 800 × 700 km^2^ and an altitudinal range of 1 to 1,520 m above sea level. Populations show an average pairwise distance of 333 ± 184 km, with a minimum and maximum of 2 and 986 km, respectively. This collection covers the environmental and ecological diversity of *C. hirsuta* in this region, and was develop for comparative studies with *Arabidopsis thaliana*. To reduce potential biases in interspecific comparisons derived from different environmental distributions or ecological niches, accessions were collected from the same (<100 m; 35%) or nearby (<50 km; 65%) locations than *A. thaliana* populations previously analyzed (Castilla et al. 2020; Arteaga et al. 2021). Genome sequences of 48 accessions have been previously described (Baumgarten et al. 2023), but additional sequences have now been obtained for the remaining 75 accessions.

A total of 81 environmental variables were obtained for *C. hirsuta* populations as previously described (Castilla et al. 2020). Briefly, climatic data of each population location were derived from the digital climatic atlas of the Iberian Peninsula (https://opengis.grumets.cat/wms/iberia/) at 1 km^2^ resolution following the climatic models described by Ninyerola et al. (2000). These include mean, maximum, and minimum temperatures, total precipitation and mean solar radiation for each month, as well as 19 bioclimatic variables calculated from monthly data (Hijmans et al. 2005). In addition, population habitat was determined in each location as the proportion of agricultural land per km^2^, which was estimated from the 48 landscape classes of CORINE Land Cover Map 2018 (http://www.idee.es). The percentage of agriculture was quantified by counting the number of 100 m grid cells belonging to any of the CORINE agricultural categories (numbers 12 to 22 including arable land, permanent crops pastures, and heterogeneous agricultural areas) per km^2^.

### Growth conditions and phenotypic analyses

Plants were grown at 21 and 26 °C to represent normal and moderately high ambient temperatures (Lippmann et al. 2019). To this end we used pots with soil and vermiculite at 3:1 proportion and growth chambers set up at 21 or 26 °C and long-day (LD; 16 h of cool-white fluorescent light, photon flux of 100 *µ*mol/m^2^ s) photoperiod. Vernalization treatment was given in a cold chamber at 4 °C, with short-day (SD; 8 h light, 16 h darkness), during 8 wk.

For analyses of trichome pattern across ontogeny, five accessions covering the Iberian geographic and genomic diversity were grown in the same experiment with a design of four complete and randomized blocks, each block containing one pot with four plants per line. The first 10 rosette leaves of each plant were collected and photographed when they were fully expanded, and trichome number (TN) and the terminal leaflet size (LS) were scored on the photographs using the image analysis software ImageJ (http://imagej.net). Leaf trichome density (TD) was quantified as the ratio between TN and LS in mm^2^. TD and TN were quantified in the adaxial and abaxial sides of leaves sampled from different plants grown in separate experiments under similar environmental conditions.

For phenotypic characterization at 21 or 26 °C, the 123 accessions were grown simultaneously in a single experiment with a design of four complete blocks with randomization, each block containing one pot with four plants per line. Trichome number (TN21, TN26) and density (TD21, TD26) were quantified on the adaxial side of leaf 6 to 7, as described above. The plasticity to temperature of TN (PTN), TD (PTD), or LS (PLS) was estimated as previously described (Valladares et al. 2006) using the following index that compares the mean values of each accession measured at both temperatures: (meanTRAIT26 − meanTRAIT21)/(meanTRAIT26 + meanTRAIT21). Hence, plasticity indexes vary between −1 and 1 (maximum plasticity with decreased or increased trait values at 26 C, respectively), whereas 0 corresponds to absence of plasticity.

### Distribution modeling and environmental analyses

*Cardamine hirsuta* distribution models were generated for the Iberian Peninsula at the species level and for the two genetic groups (IBE and BAL) analyzed in this study. Major determinants of *C. hirsuta* ecology were selected as environmental predictors of the species distribution by analyzing the pair-wise correlations among the 81 environmental variables described above (Supplementary Methods). We thus selected the following six bioclimatic and one landscape variables showing nonsignificant or low correlations (*r* < 0.7): annual mean temperature (BIO1), mean temperature diurnal range (BIO2), temperature seasonality (BIO4), mean temperature of wettest quarter (BIO8), annual precipitation (BIO12), precipitation seasonality (BIO15), and the proportion of agriculture land per square kilometer. Continuous distribution models estimating predicted habitat suitability, as well as suitability response curves, were obtained with the presence-only algorithm of Maxent version 3.4.1 (Phillips et al. 2006) within the R dismo package version 1.3-3 (https://rspatial.org/raster/sdm) (Supplementary Methods). Habitat suitability is a relative index of adequacy of the species, or the genetic groups, to the environment where it occurs (Phillips et al. 2006). However, such index is not meant to be read as an absolute probability of presence, but as how suitable a given location or grid cell is with respect to the others. Response curves quantify the effect of each environmental predictor on the model predicted suitability, namely, how suitability changes along each environmental gradient when the rest of variables are kept at their average (Phillips et al. 2006).

The spatial autocorrelation of trichome traits was analyzed using correlograms generated with the software PASSaGE version 2 (Rosenberg and Anderson 2011). For each variable, Moran’s *I* autocorrelation coefficients were calculated and plotted for 30 successive spatial intervals, coefficients ranging between 1 (positive spatial autocorrelation) and 0 (no autocorrelation). Significances of Moran's *I* values were calculated from 1,000 permutations.

The relationships between environmental variables and trichome traits were tested in the entire Iberian Peninsula region using simultaneous autoregressive models, which correct for spatial autocorrelation and lack of independence of samples (SAR; Kissling and Carl 2008). SAR is a multiple regression technique explicitly developed for spatial data, which uses generalized least squares to estimate regression parameters while including in the model an additional term for the autocorrelation matrix of the errors (Beale et al. 2010). The relationships between significant climatic variables and quantitative traits were also tested at lower geographic scale by GWR, which is also a spatially explicit explanatory regression. GWR performs a local regression test in each of the population locations by taking into account a fraction (10% to 15%) of the samples corresponding to neighboring locations selected at an optimal bandwidth distance. For each location, the selected bandwidth distance is optimized using a Gaussian spatial weighting function minimizing the Akaike information criterion (AICc). This procedure enhances the goodness-of-fit of GWR by setting the optimal number of neighboring locations to perform local regressions, as well as by controlling for border and sampling effects. All spatial regression analyses were carried out using SAM software version 3.1 (Rangel et al. 2010). Five glabrous accessions were not considered in these analyses.

### Genome sequencing and genetic structure

DNA for genome sequencing was isolated from mature leaves as previously described (Baumgarten et al. 2023). Genome sequences of 75 wild accessions from the Iberian Peninsula were generated from paired-end libraries obtained through the Max-Planck Genome Center (MPGC, Cologne, Germany) using Illumina HiSeq2000 or HiSeq3000 instruments (Illumina, San Diego) and are available at NCBI SRA under the BioProject accession number PRJNA998743. These sequences were analyzed by the Service of Bioinformatics for Genomics and Proteomics (CNB-CSIC, Madrid, Spain) together with similar sequences previously generated for 48 Iberian accessions (Baumgarten et al. 2023). SNP calling and genotyping, as well as functional annotation of each genome, were carried out following the pipelines described in Arteaga et al. (2021) (Supplementary Methods). For GWAS, only SNPs showing a minor allele frequency of five accessions (MAF ≥ 3%) were considered, providing a total of 3,281,070 informative SNPs, from which 254,468 had no missing data (Supplementary Table S2). These SNPs were located in 29,133 genes out of the 29,458 annotated open reading frames (Gan et al. 2016), and all but 171 genes contained more than one SNP. On average, genomes of the 123 Iberian accessions had one SNP every 60 bp, which corresponded to 113 SNPs per gene.

The genetic structure of the Iberian accessions was estimated using the network clustering by NJ and the PC analyses implemented in TASSEL version 5 (Bradbury et al. 2007), as well as the Bayesian model-based clustering algorithm implemented in ADMIXTURE (Alexander et al. 2009) (Supplementary Methods). Population nucleotide diversities were calculated with TASSEL, and the genetic differentiations among populations (*F_ST_*) were estimated using the R package SNPRelate (Zheng et al. 2012) with the Weir and Cockerman method.

IBD analyses were carried out by Mantel tests using PASSaGE software (Rosenberg and Anderson 2011). For this, genetic distances were measured as proportions of allele differences between pairs of accessions.

### Phenotypic and environmental GWAS

GWAS of trichome traits were carried out applying the standard mixed linear model implemented in TASSEL (Bradbury et al. 2007) on the mean values of the 118 nonglabrous accessions and the 3.3 million SNP dataset described above. The genetic kinship matrix included as covariate to control for population structure was estimated from the proportion of shared alleles (1001 Genomes Consortium 2016) using the set of 4.5 million nonsingleton high quality SNPs. To detect the most significant associations we applied a high significance threshold of −log(*P*) = 6.3, corresponding to a false discovery rate (FDR) of 0.1 after correction for multiple tests by Benjamini–Hochberg procedure. In addition, we also applied a low significance threshold of −log(*P*) = 4 to detect potential associations, as described for large SNP datasets (Togninalli et al. 2018). Given the complexity of quantitative traits and the limited population size, comparisons of GWAS results among different variables are based on the overlapping of genes instead of SNPs. The number of associated genes detected by GWAS was derived from gene locations of significant SNPs, but including the two flanking genes when SNPs were located in intergenic regions.

Broad sense heritabilities (*h^2^_b_*) of trichome traits and heritabilities explained by associated SNPs were calculated from variance components estimated by the restricted maximum likelihood (REML) method, using the general linear models (GLMs) implemented in SPSS software, version 29. The heritability of trichome variables explained by the kinship matrix was estimated by genomic best linear unbiased prediction (BLUP) as implemented in TASSEL (Bradbury et al. 2007). Genetic differentiation between groups of accessions for quantitative traits were calculated as *Q_ST_* values. Between groups (*V_B_*) and within groups (*V_W_*) variances were estimated by the REML method of variance component analysis, and *Q_ST_* was calculated as *V_B_*/(*V_B_* + *V_W_*). Main SNPs associated with TN or TD variables were tested for interactions with temperature by GLMs with repeated measurements using trait values (TN or TD, respectively) at different temperatures as the within-subject dependent variables and the SNPs as between subject factors.

EGWAS were carried out for the climatic variables most significantly associated with trichome traits using the latent factor mixed model (LFMM) (Gain and François 2021) and the 3.3 million SNP data set. In the LFMM method, the allele frequency at a locus is the dependent variable explained by a fixed environmental factor and the random effects of hidden (latent) factors representing residual levels of population structure (François et al. 2016). LFMM was applied using the R package LEA3 and lffm2 function with *K* = 2 latent factors because this is the number of genetic groups estimated in the Iberian collection. Adjusted *P* values were obtained for each SNP using the genome inflation factor method (François et al. 2016). A significance threshold of −log(*P*) = 4, corresponding to a FDR = 0.15 after correction for multiple tests by Benjamini–Hochberg procedure, was applied for detection of environmental associations.

To find *C. hirsuta* candidate genes involved in trichome development that are associated with phenotypic or environmental variables, we used a list of 155 *A. thaliana* genes known to affect trichome patterning and development, previously described (Arteaga et al. 2022). *C. hirsuta* orthologous genes were searched based on gene and protein homology, as well as on macro and microsynteny using the web-based SynMap software as implemented in the Comparative Genomics CoGe platform (Haug-Baltzell et al. 2017). We thus identified 149 *C. hirsuta* orthologues and three additional paralogues showing very high homology (>80% protein similarity) with *A. thaliana* genes. However, six *A. thaliana* genes did not detect *C. hirsuta* orthologues (Supplementary Table S9). *C. hirsuta* genes were named similar to *A. thaliana* orthologues, adding a *Ch* prefix. Similarly, to define the potential function of the most significant *C. hirsuta* genes detected by GWAS, we also searched for their *A. thaliana* orthologues, and *C. hirsuta* genes were named accordingly (Supplementary Table S8).

### *ChGL1* sequencing, phylogenetic, and expression analyses

The 2.9 kb of *ChGL1* coding region from the five glabrous accessions and two hairy lines (Hin-0 and Slc-0) was sequenced by PCR amplification of four overlapping fragments of 0.5 to 0.9 kb (Supplementary Table S10). Amplified products were sequenced using an ABI PRISM 3730xl DNA analyzer. DNA sequences were aligned using DNASTAR version 17.0 (Lasergene) and alignments were inspected and edited by hand with GENEDOC (Nicholas et al. 1997). Gene sequences are available in GenBank/EMBL databases under the accession numbers OR287788-OR287794.

The *ChGL1* relationships among the 123 Iberian accessions were determined from 2,861 SNPs identified in the genome sequences as located within the 63 kb spanning between *ChGL1* and flanking genes. These polymorphisms were used to construct a NJ tree with MEGA version 7 (Tamura et al. 2011), applying 10,000 bootstrap permutations for statistical significances.

To quantify *ChGL1* expression, plants were grown as described for phenotypic analyses, but pots contained ∼25 seeds. The 12 accessions to be compared were grown simultaneously in a single experiment, including three pots per genotype, organized in three randomized blocks (three biological replicates). After sowing, pots were placed at 4 °C and SD photoperiod for seed stratification. Thereafter, pots were transferred to a growth chamber with LD photoperiod and 21 °C, and 14- to 18-day-old rosettes were harvested. RNA was isolated using TRIzol reagent according to manufacturer's protocol (Invitrogen). Potential DNA contamination was removed by DNAse digestion and subsequent RNA purification was carried out with high pure RNA isolation kit (Roche). cDNA was synthesized from 3 *µ*g of total RNA using AMV reverse transcriptase (Invitrogen) and dT15 oligonucleotides. *ChGL1* expression was analyzed by reverse transcription-quantitative PCR (RT-qPCR) (Supplementary Table S10). The housekeeping gene *ChTIP41* (*CARHR242510*) previously described (Pérez-Antón et al. 2022), was used as endogenous control for RNA sample standardization (Supplementary Table S10). To avoid amplification differences caused by DNA polymorphisms, primers in *ChGL1* and *ChTIP41* were designed in gene regions carrying no polymorphism among the 12 *C. hirsuta* accessions analyzed. All genes were amplified with Power SYBR green mix in a 7,300 real time PCR system (Applied Biosystem) and quantified using the standard curve method. Mean and standard errors were derived from three biological replicates and four technical replicates per sample (RT-qPCR wells from the same cDNA sample).

### Other statistical analyses

Phenotypic and gene expression differences between accessions were tested by mixed general linear models including genotypes and ontogeny or temperatures as fixed effect factors, and replicates as random effect factor. These analyses were carried out with the statistical package SPSS. All statistical analyses are detailed in Supplementary Table S5.

### Accession numbers

Sequence data from this article can be found in the GenBank/EMBL data libraries under accession numbers OR287788-OR287794, and in NCBI SRA under the BioProject accession number PRJNA998743.

## Supplementary Material

kiae213_Supplementary_Data

## Data Availability

The data underlying this article are available in the article and in its on line supplementary material.

## Supplementary data

The following materials are available in the online version of this article.

**Supplementary Figure S1.** Additional information on the genetic and geographic structure of *C. hirsuta*.

**Supplementary Figure S2.** Geographic structure of *C. hirsuta* and *A. thaliana* in the Iberian Peninsula.

**Supplementary Figure S3.** Environmental diversity across *C. hirsuta* populations.

**Supplementary Figure S4.** Relationships between leaf trichome traits in *C. hirsuta*.

**Supplementary Figure S5.** Geographically weighted regressions between climatic variables and trichome traits.

**Supplementary Figure S6.** GWAS of trichome traits.

**Supplementary Figure S7.** Comparisons of genes detected by GWAS of different traits.

**Supplementary Figure S8.** Geographic and climatic distribution of polymorphisms in top genes found by phenotypic and environmental GWAS.

**Supplementary Figure S9.** Genetic diversity and geographic distribution of *GL1* in *C. hirsuta* and *A. thaliana*.

**Supplementary Figure S10.** Genetic analysis of an F_2_ (Tod-6 × Slc-0) segregating for glabrousness and *ChGL1*.

**Supplementary Table S1.** Geographic and phenotypic information of *C. hirsuta* Iberian accessions.

**Supplementary Table S2.** Genome-wide SNPs genotyped in 123 Iberian accessions.

**Supplementary Table S3.** Nucleotide diversity and genetic differentiation in *C. hirsuta*.

**Supplementary Table S4.** Parameters of *C. hirsuta* distribution models for the species and the two genetic groups.

**Supplementary Table S5.** Summary of statistical analyses.

**Supplementary Table S6.** Correlations among trichome-related traits.

**Supplementary Table S7.** Regression analyses between environmental variables and trichome traits.

**Supplementary Table S8.** Summary of GWAS of trichome pattern traits.

**Supplementary Table S9.** *A. thaliana* genes and *C. hirsuta* orthologues involved in trichome patterning and development.

**Supplementary Table S10.** Oligonucleotides used for *ChGL1* sequencing, genotyping, and expression analyses.

## Dive Curated Terms

The following phenotypic, genotypic, and functional terms are of significance to the work described in this paper:

PLS Gramene: AT4G39403
PLS Araport: AT4G39403

BIO1 Gramene: AT5G57590
BIO1 Araport: AT5G57590

BIO2 Gramene: AT2G43360
BIO2 Araport: AT2G43360

ETC2 Gramene: AT2G30420
ETC2 Araport: AT2G30420

AtMYC1 Gramene: AT4G00480
AtMYC1 Araport: AT4G00480

CPC Gramene: AT2G46410
CPC Araport: AT2G46410

EGL3 Gramene: AT1G63650
EGL3 Araport: AT1G63650

PD1 Gramene: AT2G27820
PD1 Araport: AT2G27820
