# Correction to: The trichome pattern diversity of Cardamine shares genetic mechanisms with Arabidopsis but differs in environmental drivers

**DOI:** 10.1093/plphys/kiae539

**Published:** 2024-10-17

This is a correction to: Alberto Fuster-Pons, Alba Murillo-Sánchez, Belén Méndez-Vigo, Arnald Marcer, Bjorn Pieper, Rafael Torres-Pérez, Juan Carlos Oliveros, Miltos Tsiantis, F Xavier Picó, Carlos Alonso-Blanco, The trichome pattern diversity of Cardamine shares genetic mechanisms with Arabidopsis but differs in environmental drivers, *Plant Physiology*, 2024; kiae213, https://doi.org/10.1093/plphys/kiae213

In the originally published version of this manuscript, the funding acknowledgements were incomplete. The following funding sources have been added: ‘the Max Planck Society (core grant to M.T.), and the Deutsche Forschungsgemeinschaft (DFG) (CRC TRR 341 Plant Ecological Genetics project A3 to M.T.)’.

Additionally, some of the dots in Figure 3 F should have been white in colour, rather than solid red or green. The figure has been corrected.

