# Supplementary material for: The trichome pattern diversity of Cardamine shares genetic mechanisms with Arabidopsis but differs in environmental drivers
Source: Plant Physiol. 2024 Apr 12;196(4):2730–48. doi: 10.1093/plphys/kiae213 (PMC11637488; doi:10.1093/plphys/kiae213)
Supplement: kiae213_Supplementary_Data [file kiae213_supplementary_data.zip › FusterPons_et_al_Supplementary_Data.pdf]

SUPPLEMENTARY METHODS

Distribution modeling

C. hirsuta distribution models were generated for the Iberian Peninsula at the species level and for the two genetic groups (IBE and BAL) described in this study. For the species modeling we used 834 occurrences of *C. hirsuta* from three sources: preserved specimens from natural history collections (https://doi.org/10.15468/dl.rttjj7); data from ANTHOS database (Medina Domingo and Aedo Pérez, 2021); and our own collection. To prevent potential errors derived from spatial uncertainty (Marcer et al., 2022), we only included Iberian records with a coordinate uncertainty below 707 m, which is compatible with a 1 km^2 environmental predictor resolution. In addition, only one occurrence per 1 km^2 grid cell was used to avoid possible bias in distribution models. For modeling the distribution of *C. hirsuta* genetic groups, 72 (IBE) and 51 (BAL) occurrences were used corresponding to the locations of populations with ancestry coefficients higher than 0.7 in those genetic groups (Supplementary Table S1).

The 81 environmental variables collected from sampling locations of the 123 accessions (see Materials and Methods) were analysed by principal component (PC) analyses (Supplementary Figure S3), hence showing a broad species environmental diversity, with a larger environmental space for IBE than BAL group. Major determinants of *C. hirsuta* ecology, used as environmental predictors of the species distribution, were then selected by analysing the pair-wise correlations among the 81 environmental variables. We thus selected the following six bioclimatic and one landscape variables showing non-significant or low correlations (r<0.7): annual mean temperature (BIO1), mean temperature diurnal range (BIO2), temperature seasonality (standard deviation x 100; BIO4), mean temperature of wettest quarter (BIO8), annual precipitation (BIO12), precipitation seasonality (coefficient of variation; BIO15), and the proportion of agriculture land per km^2. All variables showed substantial variation among *C. hirsuta* locations, as well as partial differentiation between both IBE and BAL groups (Supplementary Figure S3).

Continuous distribution models were obtained with the presence-only algorithm of Maxent version 3.4.1 (Phillips et al., 2006) within the R dismo package version 1.3-3 (Hijmans et al., 2020). We randomly chose 10,000 points across the Iberian Peninsula to represent the environmental background available to the species and extracted their predictor variable values using the function extract from the R raster package (Hijmans, 2021). If a point had a “nodata” value we used the value of its closest 1 km^2 grid cell within a radius of 10 km instead. We used Maxent with autofeatures and tested three values (1, 2 and 5) for the Maxent's betamultiplier regularization parameter to account

for possible overfitting (Merow et al., 2013). In addition, we used 5-fold cross-validation to measure model performance and selected the best model as that with the highest mean test AUC value (area under the receiver-operator curve). The contribution of environmental variables to each distribution model was estimated by Maxent as normalized percentage drop in training AUC when the values of a given variable are permuted and the model is re-evaluated. The predicted suitability was estimated in each model as a relative index of adequacy of the species, or the genetic groups, to the environment where it occurs (Phillips et al., 2006). Response curves were obtained to quantify the effect of each environmental predictor on the model predicted suitability, namely, how suitability changes along each environmental gradient when the rest of variables are kept at their average (Phillips et al., 2006). For the species and genetic group models, we used the whole set of variables since we wanted to compare variable responses between them.

Genome sequencing and genetic structure analyses

The new genome sequences of 75 wild Cardamine accessions from the Iberian Peninsula were analysed together with sequences previously generated for 48 Iberian accessions (Baumgarten et al., 2023). Sequences from each accession were aligned against Oxford reference strain (Gan et al., 2016) using IMR-DENOM-IRISAS (Song et al., 2018) and alignment quality was analysed with the Qualimap version 2.2.1 software (Okonechnikov et al., 2016). Sequencing depths and coverages were calculated with bcftools version 1.6 (Li, 2011), thus estimating an average sequencing depth for the 123 genomes of 17x ± 3.2, and a mean coverage of 90% of the nuclear genome with at least 5 reads.

The software bcftools was used for variant calling on each individual alignment, but indels were filtered out to maintain only SNPs in the variant call format (VCF) files (Danecek et al., 2011). SNPs were genotyped only at genomic positions with a minimum depth of 5 reads. All individual variant files were then merged using bcftools, into a single multi-sample VCF file, which contained 10.96 million nuclear SNPs. This file was filtered to remove low quality SNPs, keeping 6.27 million binary SNPs displaying two ACTG classes (reference and alternative, or two different alternatives), with coherent heterozygosity (heterozygosity lower than 3% and corresponding to the two alternative ACTG classes detected in that position) and at least 55% non-missing genotyped accessions. Heterozygous calls in these SNPs were rescored to the major frequency allele. For GWA analyses, five glabrous accessions and their private SNPs were removed and the SNPs were further filtered to keep only those with a minor allele

frequency equal or higher than 3%. Thus, we generated a final VCF file containing 3,281,070 SNPs for GWA studies in 118 Iberian accessions, with an average missing information per SNP of 8.5%. SNPs were functionally annotated with snpEff tool version 4.3t (Cingolani et al., 2012) using the default parameters and Cardamine genome annotation version 1.0 (Gan et al., 2016) (Supplementary Table S2).

The population structure of the 123 Iberian accessions was estimated by the neighbour-joining (NJ) clustering method implemented in TASSEL version 5 (Bradbury et al. 2007) using two SNP datasets: the 4.5 million non-singleton SNPs (Supplementary Table S2) and the 343,364 non-singleton SNPs with no missing data. Pairwise distances among all accessions were calculated as the proportion of different SNPs and both data sets rendered nearly identical results. On average, the 123 accessions showed a genetic distance of 0.20, and only four pairs displayed genetic distances lower than 0.05 (Supplementary Figure S1). These closely related pairs of accessions were estimated to differ in 2398 to 5358 high quality SNPs and their geographic distances varied from 2 to 512 km (Supplementary Figure S1C).

The population structure of the Iberian accessions was also estimated by the Bayesian model-based clustering algorithm implemented in ADMIXTURE (Alexander et al., 2009) using the same two datasets of non-singleton SNPs used for NJ analysis. For each SNP data set, the algorithm was run 20 times for each K value from K=2 to K=10. The most likely K value was estimated as that showing the lowest cross-validation error (Alexander et al., 2009), and differences between the cross-validation error of successive K values were tested by the nonparametric Wilcoxon test for two related samples (two-sided $P<0.05$). Thus, a final K=2 was estimated (Supplementary Figure S1D), which was supported by a high similarity among the ancestry membership matrices from different runs of the same K=2 value ($H'=0.99$). The average symmetric similarity coefficient H' among runs and the average matrix of ancestry membership proportions were calculated with CLUMPP (Jakobson and Rosenberg, 2007). The two SNP datasets provided nearly identical results, with an optimum of two genetic groups (K=2) and a high correlation ($r=0.99$; $P<0.001$) between the ancestry membership coefficients estimated for both data sets.

Population structure was also estimated by principal component (PC) analysis, as implemented in TASSEL version 5.2 (Bradbury et al., 2007). The same two datasets of non-singleton SNPs described above were analysed. The first PC (PC1) accounted for 29.9-30.6% of the variance, whereas the second PC (PC2) only explained 2.8-2.9% of the variance, depending on the SNP dataset. Values of the first two PCs showed correlations of 0.97-0.99 between both SNP data sets, and PC1 values derived from both

SNP sets detected the same two genetic groups highly differentiated. Comparisons of NJ, PC and ADMIXTURE results, based on the pairwise gentic distances, the largest ancestry coefficients and the PC1 scores of each sample, showed that all accessions were assigned to the same genetic group by both methods.

REFERENCES

Alexander DH, Novembre J, Lange K (2009) Fast model-based estimation of ancestry in unrelated individuals. Genome Res **19:** 1655-1664

Baumgarten L, Pieper B, Song B, Mane S, Lempe J, Lamb J, Cooke EL, Srivastava R, Strutt S, Zanko D, Casimiro PG, Hallab A, Cartolano M, Tattersall AD, Huettel B, Filatov DA, Pavlidis P, Neuffer B, Bazakos C, Schaefer H, Mott R, Gan X, Alonso-Blanco C, Laurent S, Tsiantis M (2023) Pan-European study of genotypes and phenotypes in the Arabidopsis relative *Cardamine hirsuta* reveals how adaptation, demography, and development shape diversity patterns. PLoS Biol **21:** e3002191

Bradbury PJ, Zhang Z, Kroon DE, Casstevens TM, Ramdoss Y, Buckler ES (2007) TASSEL: software for association mapping of complex traits in diverse samples. Bioinformatics **23:** 2633-2635

Cingolani P, Platts A, Wang le L, Coon M, Nguyen T, Wang L, Land SJ, Lu X, Ruden DM (2012) A program for annotating and predicting the effects of single nucleotide polymorphisms, SnpEff: SNPs in the genome of Drosophila melanogaster strain w1118; iso-2; iso-3. Fly (Austin) **6:** 80-92

Danecek P, Auton A, Abecasis G, Albers CA, Banks E, DePristo MA, Handsaker R, Lunter G, Marth GT, Sherry ST, McVean G, Durbin R, Group GPA (2011) The variant call format and VCFtools. Bioinformatics **27:** 2156

Gan X, Hay A, Kwantes M, Haberer G, Hallab A, Ioio RD, Hofhuis H, Pieper B, Cartolano M, Neumann U, Nikolov LA, Song B, Hajheidari M, Briskine R, Kougioumoutzi E, Vlad D, Broholm S, Hein J, Meksem K, Lightfoot D, Shimizu KK, Shimizu-Inatsugi R, Imprialou M, Kudrna D, Wing R, Sato S, Huijser P, Filatov D, Mayer KF, Mott R, Tsiantis M (2016) The *Cardamine hirsuta* genome offers insight into the evolution of morphological diversity. Nat Plants **2:** 16167

Hijmans RJ (2021) raster: Geographic Data Analysis and Modeling. R package version 3.4-13. https://rspatial.org/raster/sdm

Hijmans RJ, Phillips S, Leathwick J, Elith J (2020) dismo: Species Distribution Modeling. R package version 1.3-3. https://rspatial.org/raster/sdm

Jakobsson M, Rosenberg NA (2007) CLUMPP: a cluster matching and permutation program for dealing with label switching and multimodality in analysis of population structure. Bioinformatics **23:** 1801-1806

Li H (2011) A statistical framework for SNP calling, mutation discovery, association mapping and population genetical parameter estimation from sequencing data. Bioinformatics **27:** 2987-2993

Marcer A, Chapman AD, Wieczorek JR, Picó FX, Uribe F, Waller J, Ariño AH (2022) Uncertainty matters: ascertaining where specimens in natural history collections come from and its implications for predicting species distributions. Ecography**:** e06025

Medina Domingo L, Aedo Pérez C (2021) CSIC-Real Jardín Botánico-Anthos. Sistema de información de las plantas de España. Real Jardín Botánico (CSIC)

Merow C, Smith MJ, Silander JA (2013) A practical guide to MaxEnt for modeling species' distributions: what it does, and why inputs and settings matter. Ecography **36:** 1058-1069

Okonechnikov K, Conesa A, García-Alcalde F (2016) Qualimap 2: advanced multi-sample quality control for high-throughput sequencing data. Bioinformatics **32:** 292-294

Phillips SJ, Anderson RP, Schapire RE (2006) Maximum entropy modeling of species geographic distributions. Ecological Modelling**:** 231-259

Song B, Mott R, Gan X (2018) Recovery of novel association loci in *Arabidopsis thaliana* and *Drosophila melanogaster* through leveraging INDELs association and integrated burden test. PLoS Genet **14:** e1007699

SUPPLEMENTARY FIGURES

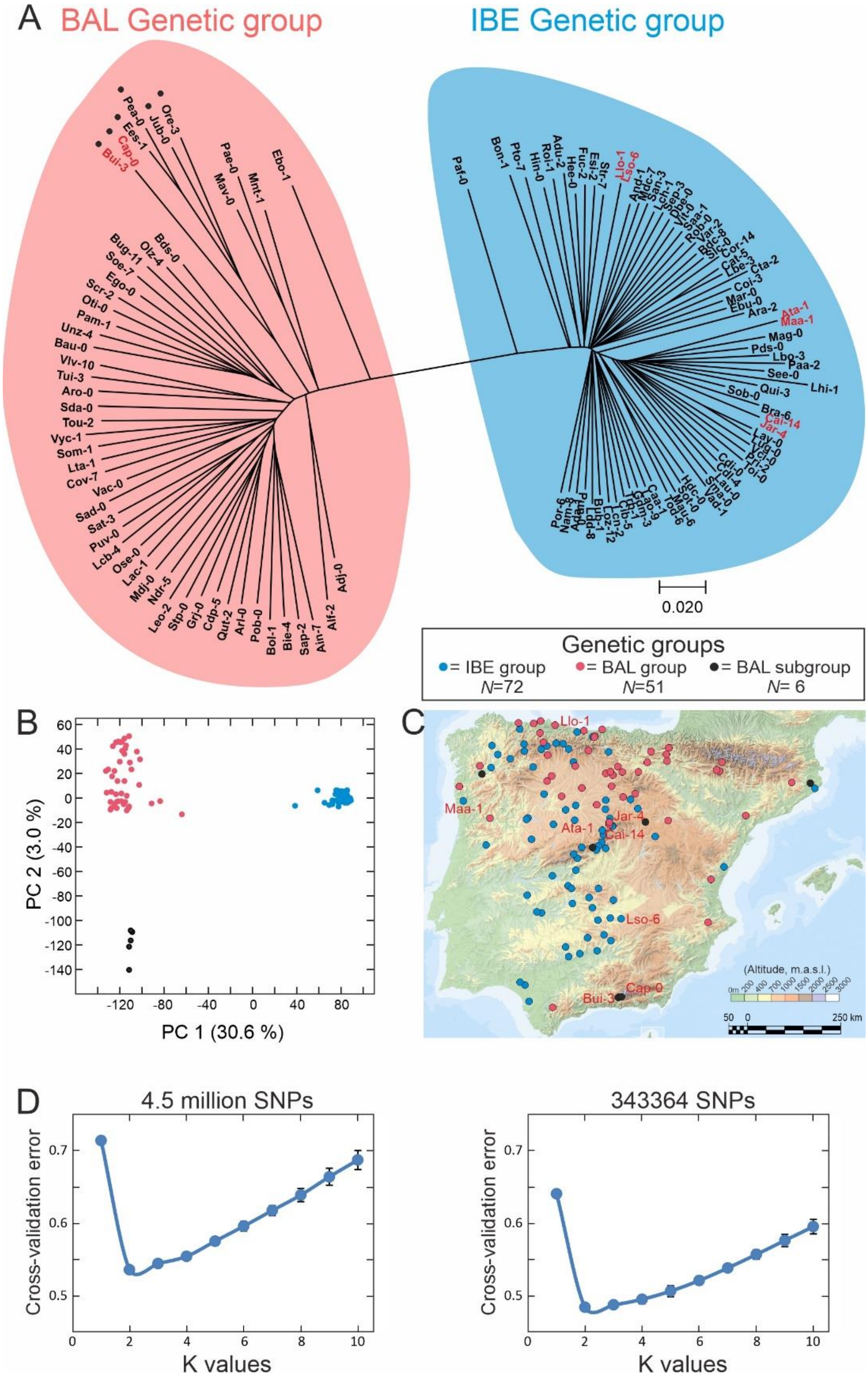

Supplementary Figure S1. Additional information on the genetic and geographic structure of *C. hirsuta* in the Iberian Peninsula. A) Neighbor-joining (NJ) tree showing the two highly differentiated genetic groups named as Iberian (IBE) and Balkan (BAL) clusters. Scale corresponds to genetic distances calculated as proportions of different

single nucleotide polymorphisms (SNPs). B) Scatter plot displaying the principal component (PC) analysis of the 123 genotypes. NJ and PC analyses was carried out using 343,364 non-singleton SNPs with no missing data segregating in the 123 accessions. Branch length is a measure of genetic distance quantified as the proportion of different alleles. C) Geographic distribution of the IBE and BAL genetic groups detected by NJ, ADMIXTURE and PC analyses. In A and C) the names of the four pairs of highly related accessions (genetic distances < 0.05) are colored in red, whereas the six BAL accessions showing low PC 2 values are depicted as black dots. D) Plots showing cross-validation errors estimated with the Bayesian model-based clustering algorithm of ADMIXTURE for different number of ancestral populations (K values). For each K value, the mean ± standard deviation of 20 ADMIXTURE runs is shown. Left panel was obtained using the 4.5 million non-singleton SNPs, whereas right panel displays the results using the 343,364 non-singleton SNPs with no missing data. In both ADMIXTURE analyses, the lowest cross-validation errors were obtained for K=2.

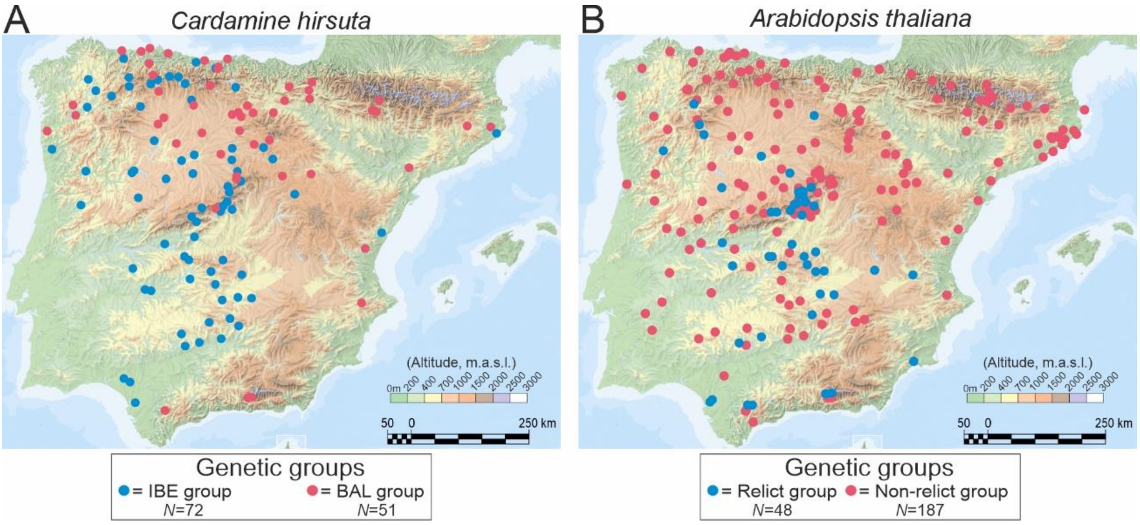

Supplementary Figure S2. Geographic structure of *Cardamine hirsuta* and *Arabidopsis thaliana* in the Iberian Peninsula. A) Geographic distribution of 123 populations of *C. hirsuta* classified in the two highly differentiated genetic groups, Iberian (IBE) and Balkan (BAL), identified in the Iberian Peninsula in this study. B) Geographic distribution of 235 populations of *A. thaliana* assigned to the two major genetic groups, Relict and Non-relict, previously described in the Iberian Peninsula (1001 Genomes Consortium, 2016; Arteaga et al., 2021).

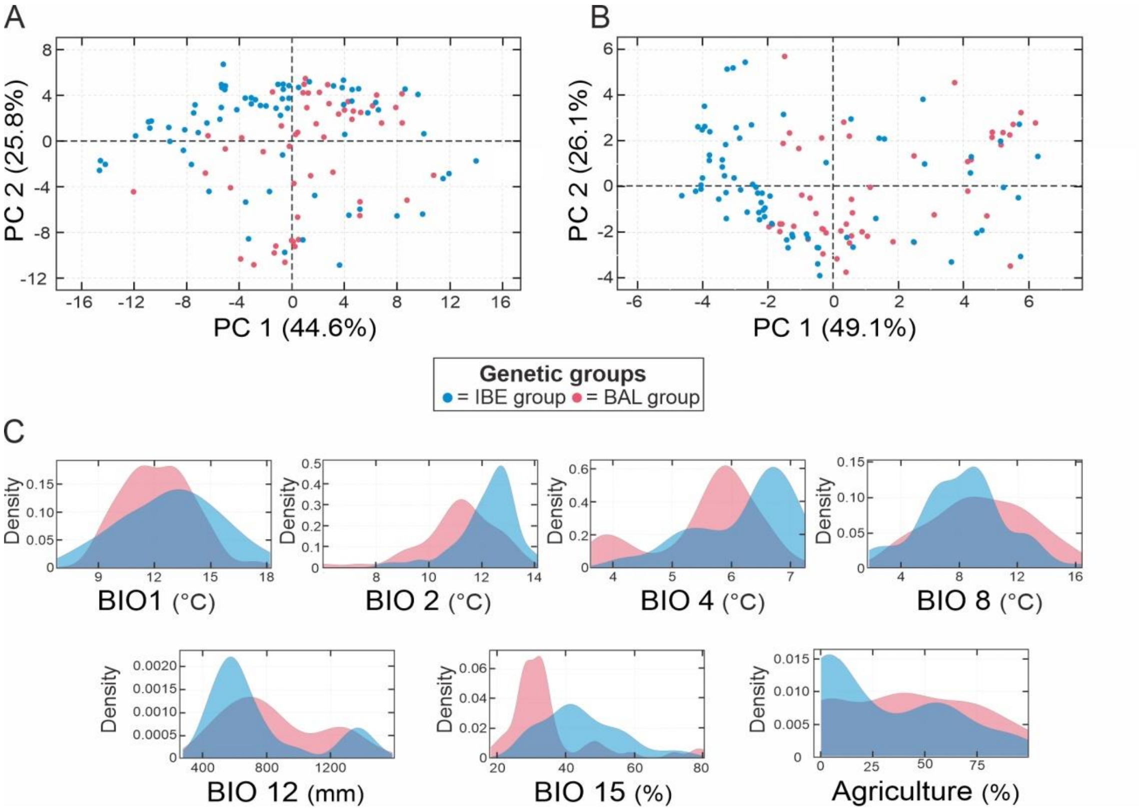

Supplementary Figure S3. Environmental diversity across *C. hirsuta* populations. A-B) Principal component (PC) analysis of 81 environmental (A) or the 19 bioclimatic (B) variables at the locations of the 123 Iberian accessions under study. C) Density plots showing the distributions of the seven environmental variables used to generate the distribution models of *C. hirsuta* at the species and genetic group levels. Populations belonging to Iberian (IBE) and Balkan (BAL) groups are shown in different colors as described in figure legends. BIO1: annual mean temperature; BIO2: mean temperature diurnal range;BIO4: temperature seasonality (standard deviation x 100); BIO8: mean temperature of wettest quarter; BIO12: annual precipitation; BIO15: precipitation seasonality (coefficient of variation); Agriculture: the proportion of agriculture land per km^2.

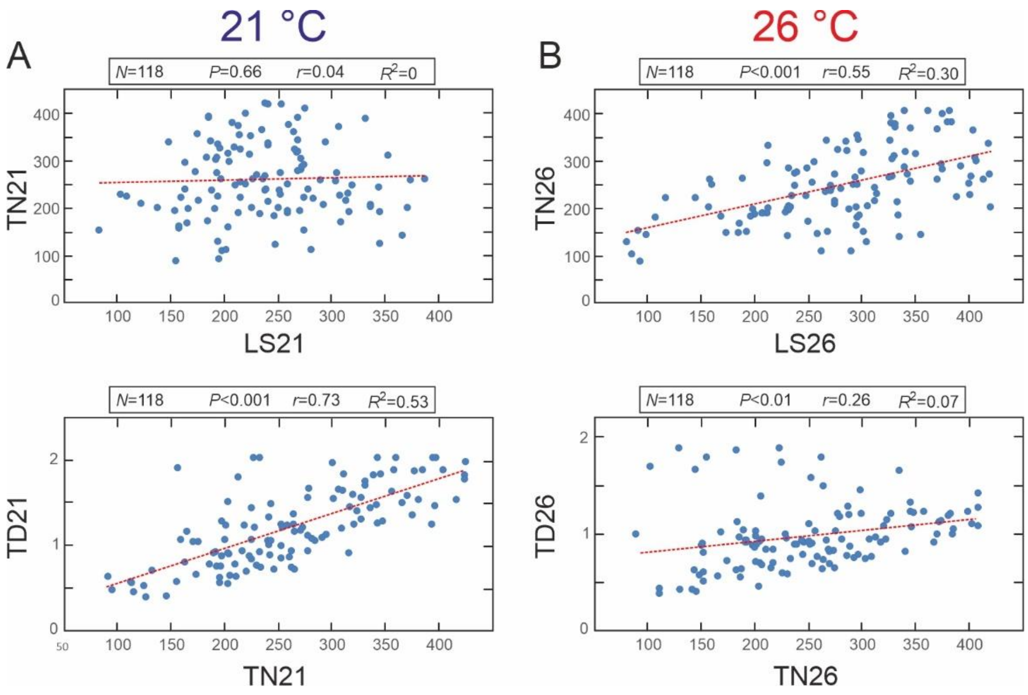

Supplementary Figure S4. Relationships between leaf trichome traits in *C. hirsuta*. A) Relationship between trichome number (TN21) and terminal leaflet surface (LS21) or trichome density (TD21) measured at 21 °C. B) Relationship between trichome number (TN26) and terminal leaflet surface (LS26) or trichome density (TD26) measured at 26 °C. In each panel, the upper legend indicates the number of samples (*N*), the statistical significance (*P*) of the Pearson correlation coefficient (*r*) and the explained variance in the regression model (R^2).

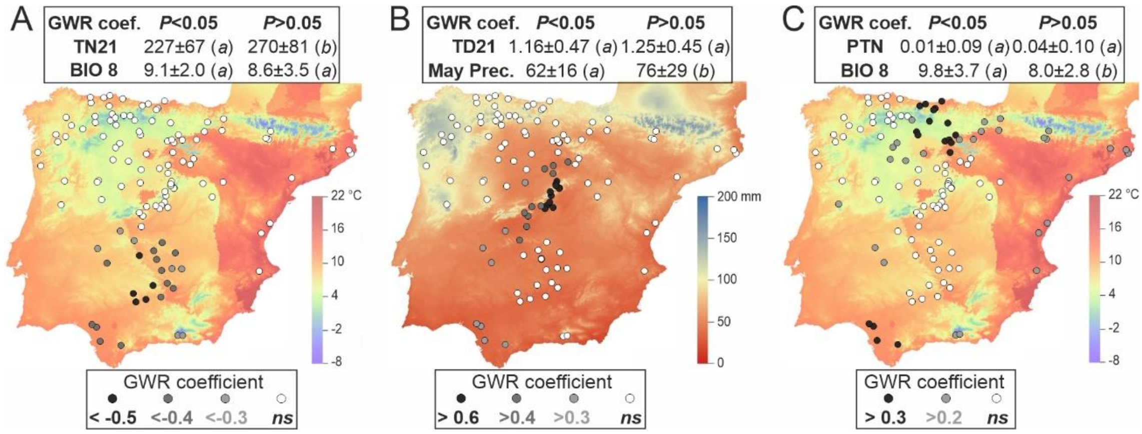

Supplementary Figure S5. Geographically weighted regression (GWR) analyses between climatic variables and trichome traits. A) GWR analysis between mean temperature of wettest quarter (BIO8) and trichome number measured at 21 °C (TN21). B) GWR analysis between May precipitation (May Prec) and trichome density at 21 °C (TD21). C) GWR analysis between BIO8 and the plasticity of trichome number (PTN). Panels show climatic maps including the GWR standard coefficients estimated at each location and depicted with different colors according to the lower legends. In the upper box of panels, mean values ± standard deviation of trichome and climate variables are shown for locations with significant or non-significant GWR coefficients (indicated as *P*<0.05 and *P*>0.05, respectively). Differences between both types of locations for these variables were statistically tested by general linear models; the same or different letters indicate non-significant or significant differences (*P*<0.05).

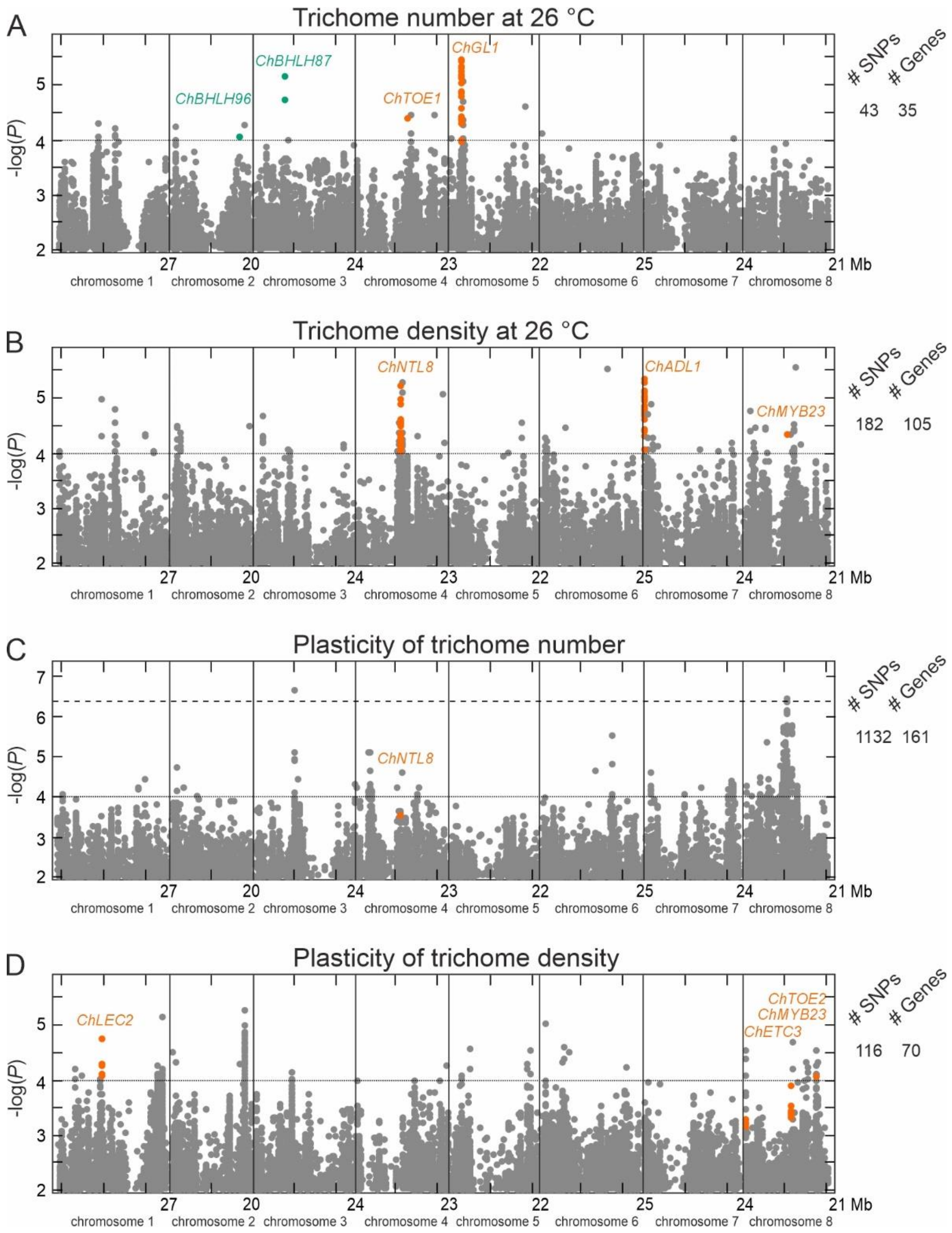

Supplementary Figure S6. GWAS of trichome traits. (A-D) Manhattan plots for leaf trichome number (TN26) (A) and density (TD26) (B) at 26 °C, as well as for the plasticity of trichome number (PTN) (C) and density (PTD) (D) to temperature, across the eight *C. hirsuta* chromosomes. Horizontal black dotted and dashed lines indicate significance thresholds of -log(*P*)=4 and FDR=0.1 after Benjamini-Hochberg correction for multiple tests, respectively. Orange and bluish green colored dots match single nucleotide polymorphisms (SNPs) with -log(*P*)>4 that are located on *C. hirsuta* orthologues or homologues, respectively, of known *A. thaliana* trichome related genes; the names of these genes are included in each panel. Trichome candidate genes associated with -log(*P*)=3 are also included when the same gene appears as associated at -log(*P*)=4 for another trait.

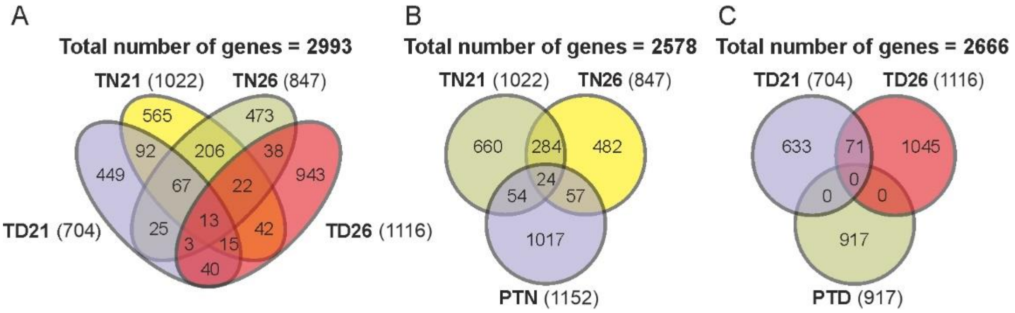

Supplementary Figure S7. Comparisons of genes detected by GWAS of different traits. Venn diagrams show the number of associated genes shared by: A) leaf trichome number and density at 21 and 26 °C (TN21, TN26, TD21, TD26); B) trichome number traits and plasticity (TN21, TN26, PTN); C) trichome density traits and plasticity (TD21, TD26, PTD). Associated genes were selected at -log(*P*)=3 threshold.

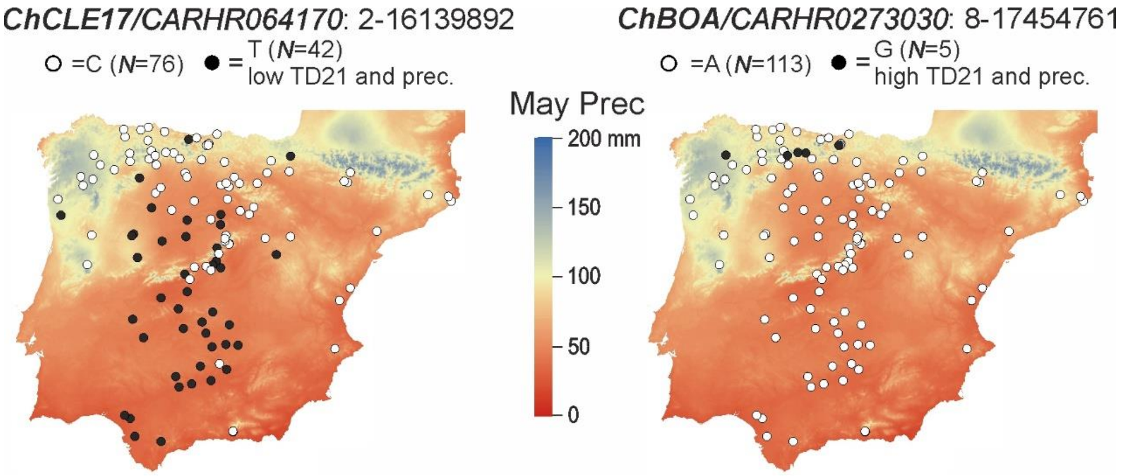

Supplementary Figure S8. Geographic and climatic distribution of polymorphisms in top genes found by phenotypic and environmental GWAS. Panels show the allele distributions of SNPs in two genes associated with May precipitation and trichome density at 21 °C (TD21), gene names and SNP positions appearing in the upper part of panels. In each map, the minor allele is depicted as black dots and the effects of the associations with TD21 and precipitation are indicated in the legend.

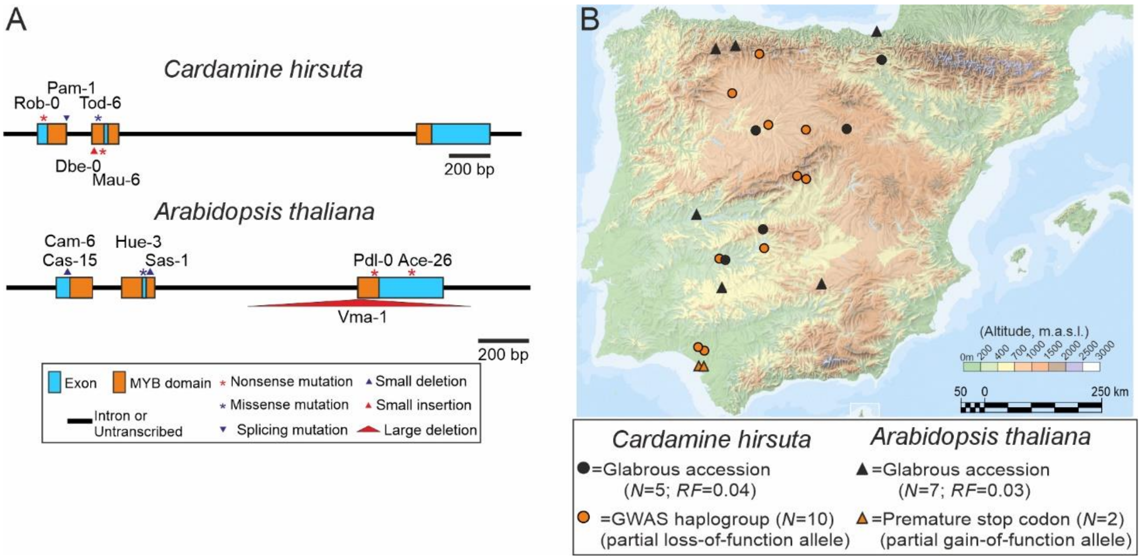

Supplementary Figure S9. Genetic diversity and geographic distribution of *GL1* in *Cardamine hirsuta* and *Arabidopsis thaliana* from the Iberian Peninsula. A) Different *GL1* loss-of-function (null) mutations causing glabrousness in *C. hirsuta* and *A. thaliana*. Information on null mutations comes from this study (for *C. hirsuta*) or Arteaga et al., 2022 (for *A. thaliana*). B) Geographic distribution of Iberian accessions carrying different alleles affecting the function of *GL1* in *C. hirsuta* and *A. thaliana*. *GL1* functional alleles are classified as complete (null) loss-of-functions producing glabrousness; partial loss- or gain-of-function alleles causing reduced or increased trichome density, respectively. The partial loss-of-function allele of *C. hirsuta* corresponds to the GWAS haplogroup found in this study and is caused by unknown regulatory mutations. The partial gain-of-function allele of *A. thaliana* is caused by a premature stop codon producing a truncated protein that lacks the last three aminoacids (Arteaga et al., 2021). The absolute (*N*) and relative frequencies (*RF*) of null alleles are estimated from the 123 *C. hirsuta* samples of this study and from the 235 *A. thaliana* samples described in Arteaga et al., 2021 (see Supplementary Figure S2 for geographic distribution of all samples from both studies).

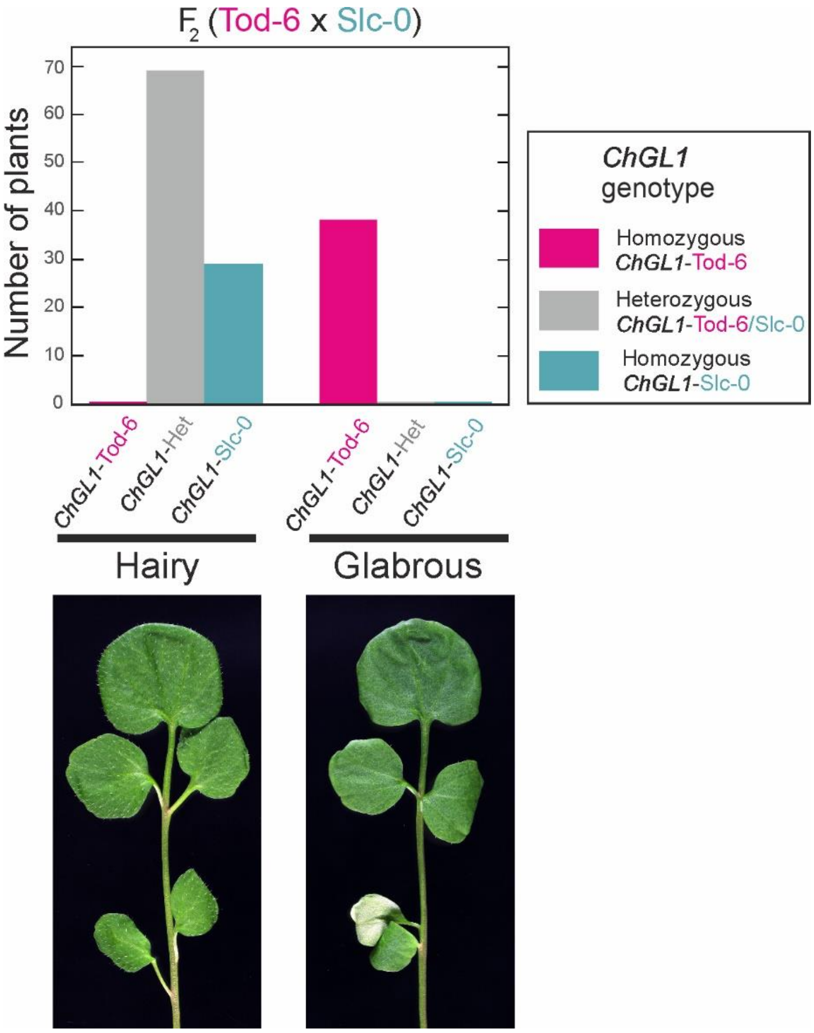

Supplementary Figure S10. Genetic analysis of an F_2 (Tod-6 x Slc-0) segregating for glabrousness and *ChGL1*. The bar diagram shows the frequency of plants classified according to the qualitative trait (glabrous/hairy) and the *ChGL1* allele. A total of 136 plants were analysed phenotypically for their trichome pattern and genotyped for the *ChGL1*-Tod-6 missense mutation.
