# Supplementary material for: The trichome pattern diversity of Cardamine shares genetic mechanisms with Arabidopsis but differs in environmental drivers
Source: Plant Physiol. 2024 Apr 12;196(4):2730–48. doi: 10.1093/plphys/kiae213 (PMC11637488; doi:10.1093/plphys/kiae213)
Supplement: kiae213_Supplementary_Data [file pp.00219.2024-s04.pdf › pp.00219.2024-s04.pdf]

Plant Physiology®

Trichome pattern diversity of Cardamine shares genetic mechanisms with Arabidopsis but differs in environmental drivers

Alberto Fuster-Pons, Alba Murillo-Sánchez, Belen Mendez-Vigo, Arnald Marcer, Bjorn Pieper, Rafael Torres-Pérez, Juan Carlos Oliveros, Miltos Tsiantis, Xavier Pico, and Carlos Alonso-Blanco

Review Timeline:

Submission Date:	22-Nov-2023
Editorial Decision:	11-Jan-2024
Resubmission Received:	08-Feb-2024
Editorial Decision:	25-Mar-2024
Revision Received:	29-Mar-2024
Accepted:	29-Mar-2024

11-Jan-2024

Dr. Carlos Alonso-Blanco
Consejo Superior de Investigaciones Cientificas
Plant Molecular Genetics
Madrid, Sp N/A
Spain

RE: Trichome pattern diversity of Cardamine shares genetic mechanisms with Arabidopsis but differs in environmental drivers

Dear Dr. Carlos Alonso-Blanco:

Thank you for giving us the opportunity to review your manuscript for publication in the Plant Physiology. All three expert reviewers and I agree that your manuscript focuses on interesting points on convergent evolution and trichome plasticity. However, there are a number of points the reviewers have shared and addressing the reviewers' comments in a revised submission will provide some of the missing justifications needed to fully align the authors' findings with their inferences. As these concerns are of some gravity and will entail substantial changes to the manuscript, you are likely to need additional time to address them. I must therefore reject the manuscript in its present form. Nonetheless, if you are able to address the points raised in review, I would highly welcome your resubmission of a manuscript that incorporates your responses.

Please note that any resubmission will be treated as a new entry to the system and is likely to go out for review.

If you do have any queries or concerns, you are welcome to contact me in the first instance.

Thank you again for allowing Plant Physiology to review your work. We look forward to hearing from you soon.

Sincerely,

Board of Editors, Plant Physiology

-- Reviewer comments:
Reviewer #1 (Comments for the Author):

This is an interesting study of trichome variation among accessions of Cardamine hirsuta from the Iberian peninsula. The authors provide very interesting data on the diversity of trichome phenotypes in these accessions, the plasticity of these phenotypes in response to temperature, the correlation of trichome phenotypes with environmental variables, and the genes that are likely responsible for the observed variation. The manuscript is well-written and to the point. I do have a few questions, however.

Most importantly, while I find the identification of specific genes with trichome density and number to be convincing, rounding up the usual suspects plus a few new ones, I am rather less convinced by the GWAS attempting to show a correlation between individual genes and May precipitation in Fig. 4C. While an FDR>0.1 may be a reasonable significance threshold when one has a sharply defined genomic region including many SNPs. But when only a handful of SNPs cross that threshold, and particularly when they are no narrowly focused on a specific genomic region as a distinct peak (as with the GL1 region in 4C), then this seems like a pretty soft threshold.

Second, I think that insufficient attention was given to the possibility that trichome density variation may be due as much to drift as to selection. Given the frequency with which glabrous varieties have arisen independently in Cardamine, Arabidopsis and related species, I'm not sure that I would describe this as "convergent evolution" (line 530, etc.). It seems to me that the observed variation might be best described by a balance between weak positive selection for trichomes and drift towards loss of function.

Third, I have to admit that I had a hard time interpreting Fig. 3G and H, and I think this could be explained better for a more general audience. In particular, I didn't understand what is meant by "the 10-15% neighboring populations." It was also not clear to me why the box over these two panels includes both P<0.05 and P>0.05. The significance threshold should be enough, unless I am missing something.

Finally, in discussions of trichome function, I think the authors should cite the Mauricio and Rausher 1997 paper in Evolution, which was the first paper that I am aware of that tested trichome function in a ecology field experiment.

Reviewer #2 (Comments for the Author):

The shared genetic control or or 'parallelism' between related species for similar traits is of keen interest. This is generally described as convergent evolution when different alleles are under selection, rather than parallel evolution when the same alleles are selected in different populations. Shared environmental response is also important.

Here the authors genotyped and phenotyped trichomes 123 wild Cardamine across multiple temperatures. The same gene Glaborous was identified with null alleles and quantitative trait locus using GWAS in Cardamine as in known for Arabidopsis, confirming convergent evolution in the same gene within and between species. Environmental responses differed however and other loci were identified.

This highly

Excellent background introduction on molecular control, development and natural variation among trichome patterns in plants, including correct use of convergent evolution. I recommend GWAS, rather than GWA studies. And consider P-GWAS for measured traits, and E-GWAS for environments from collection location.

The study sample contains key genetics, trait, and environmental variation enabling dual investigations of how gene variants control quantitative (potentially) adaptive traits AND how gene variants are controlled by, or filtered by, the environment. When this is done in multiple species, discoveries like
"opposite climatic associations than A. thaliana, supporting that this trait might be involved in partly distinct environmental adaptations in the two plants."

This is rather unexpected for adaptive traits in related (ecologically swappable) species. Could it be due to differential geographic structure? Might another there be another environment (water vs temp?) with stronger effect that is differentially correlated with trichomes? This is not easy to test, perhaps some permutations of traits and environments with locations could be a type of null hypothesis [this is outside the scope of this paper but just something for the authors to consider]

Technical issues.
123 wild accessions is not a small sample side, but pretty good considering they from different locations? What do bioclim PCs look like for this sample set? Much spread in environmental space?

Both analyses found two highly diverse and differentiated genetic
2 groups (FST=0.23..) is rather high, but not unusual for inbreeding lines.. This is considered a deeper speciation levels of separation in largely outcrossing species, where we wouldn't expect structure to be able to accommodate levels of differentiation.

Fig1 is a huge and strong dataset! IBD [panels DEF] assub geographic distance is linear in km, but there is certainly isolation by mountains. The authors might consider rather geographic resistance surfaces, that extend the very beautiful environmental niche mappings among subspecies (Fig1H). In Fig1F, the 2 groups of pink points, I worry about the 5 BAL populations -140 Eigenvector2 in Fig1B. Where are they on the map? New studies could expand on this region for sampling, as well as the admixed populations. I would like to see a population sample dendrogram just to identify and likely remove 1 of a pair of closely related individuals, or recent hybrids, eg 4 pairs with 0 genetic distance are not genetically distinct individual samples, < 5% might be recently hybrids not adding resolution to GWAS.

Slightly reducing this sample size won't hurt association mapping as these samples are not independent, but will the reduce bias they would create..

I would show the 123 environmental points from sampling locations on Fig 1 I & J, as it looks a bit too smooth. The distribution of Agriculture % is very interesting. How was this calculated? %Ag/km^2, is that annual cropped land only? Or are perennial pastures included? Could you add the % var that each bioclim +Ag contributed to suitability color in Fig 1 G&H?

4.5M SNPs is a relatively large number? What is the extent of LD? [first remove related samples]. Fig 5b, looks like an extensive LD region over 60kb of elevated associated SNPs, this is difficult to confirm a gene, but also a larger target for trait-environmental selection, eg supergene..

Fig 2 is equally powerful, true multitrait developmental phenomics approach! Ontogeny is so important and difficult to consider. Trichome number and size of largest leaflet were used to determine trichome density (density has lower heritability), but trichome number and leaflet size may covary and be relate with genotype. Can you show a scatter plot of trichome number and leaf size for density and consider a residual trait trichome number per size expected deviation rather than a density ratio? You might have more power with using leaflet size as a covariate for trichome number than trichome density, but the results are very clear and these are related traits identifying the same genes in practice. Table1 shows overall very high heritability and major loci effects beyond kinship (that was not unexpectedly large given major structure groups)

The plasticity ratio isn't great statistically. Best to fit GxE for QTL-SNPs using measured data. This isn't critical however.

There is very good support for GL1's role in controlling trichome traits, multiple alleles, loss of function and quantitative variation all elegantly confirmed by crossing and expression analysis. Even though the LD association is broad, which is typical of inbreeding plants and improves the detection power, the causal variants should be believed, even if the direct link to environmental selection is a little broader (locus level and still important)..

May precipitation seemed to correlate with trichome number. I'm less familiar with geographically weighted regression, but seems appropriate. Moisture vs temperature are hard to disentangle as the co vary with geography.

Testing the SNP x climate variation and SNP x trichome trait variation in GWAS is a super powerful way to get at the causal trait and environment links. There is some overlap with genes identified in both scans, suggesting the May precip variable as linked with trichome genetics. I didn't see a formal enrichment test if SNPs were more common than expected, but they are likely to be. Eg 17 genes in 11 regions overlap

Distinct environmental trichome responses for A thaliana vs C hirsuta is intriguing, a caveat being the focus on Col and Ler strains of A thaliana. Might different responses to seasonal precipitation be related to seasonality of flowering? Does C hirsuta also have rapid cycling stage?

This is a very powerful, well designed and elegant study, covering all the genotype, phenotype and environmental basis to discovery new genes and novel alleles of known genes allowing comparisons among species. It is worthy of a top tier journal!

All the best
Justin Borevitz

Reviewer #3 (Comments for the Author):

The authors established a new genetic data set of Cardamine hirsuta populations in the Iberian Peninsula and explored local adaptation signatures, primarily focusing on leaf-related phenotypes, including trichome numbers. They conducted comprehensive analyses of environmental adaptation utilizing climate and genetic data, and compared adaptation features between species at the population level. The results demonstrated convergent evolution in trichome density, but also independent environmental adaptations are going on. Their approach delves into the deeper evolutionary processes that comparative genomics alone cannot fully reach.

Comments
1. I have high expectations for the potential of this approach; however, I'm somewhat disappointed by what appears to be a superficial comparison of the evolutionary processes related to the genetic architecture underlying trichome number/density adaptation. As mentioned in the abstract, "the degree of parallelism in the genetic mechanisms underlying this diversity and its environmental drivers in different species remains unclear." Hence, a demonstration of parallel changes in genetic basis is crucial, at least through the detailed comparison to A. thaliana (i.e., genetic structure, allelic distribution, and natural selection).

2. Understanding environmental adaptation is the main topic of this study, and Fig 3B highlights strong genetic environment interaction (G x E) in all traits. The dissection of loci related to GxE may help in interpreting the genetic architecture and the geographic distribution of alleles. One related study in A. thaliana is below. They analyzed G x E in FLC using GWAS.

Sasaki et al., (2015) "Missing" G x E Variation Controls Flowering Time in Arabidopsis thaliana. PLOS Genetics 11(10): e1005597.

3. Regarding the genetic architecture, how much phenotypic variation can be explained by ChGL1 genetic variation? The expression of ChGL1 (cis-regulation?) explains a trichome density of more than 80%. But what is the cause of the large expression variation? (Fig 5 E&F). This scenario looks similar to the regulation of flowering time by FLC in A. thaliana population. Are the identified GWAS peaks associated with multiple haplotypes, is the ChGL1 expression regulated by other alleles identified in GWAS, or genetic background (polygenic)?

4. For Fig 1A, the explanation of why the authors used K=2 (from their previous Pan-genome paper?) is needed. Also, in the map, which lines are admixture that the Balkan group introgressed?

5. For Fig I &J, if my understanding of the model is correct, the suitability of Bio1 in all populations appears to be almost the average of the two populations. However, the observed pattern differs significantly from both. Could the authors provide an explanation for this inconsistency?

6. If the authors could superimpose the allelic distributions of A. thaliana causing changes in trichome density phenotypes in Fig 5 A and D (or just add next to the panel for C. hirsuta), it could help the interpretation of the "parallel evolutionary process" of adaptive traits on a fine scale.

7. L369: The author mentioned that ChCLE17 and ChBOA alleles were also associated with the precipitation. Are the alleles identical between GWAS for the phenotype and climate variables?

L404 I didn't get the reason clearly why the authors concluded it. Could the authors explain in detail?

Minor comments
- L136-137: Including information about the mating system (self-pollination) would be helpful for readers.
- L188-190: Could you rephrase this part? It is unclear what the authors mean.
- Fig1 I & J: Please add explanations of 'suitability' in the main text and M&M
- L220: To me, the peak seems at "four leaves" rather than "first five leaves" in Fig 2C
- L538: It might be valuable to include a citation for the recent study.
Xuan et al., Xuan, Lijie, et al. "Genome-wide association study reveals new genes involved in leaf trichome formation in polyploid oilseed rape (Brassica napus L.)." Plant, Cell & Environment 43.3 (2020): 675-691

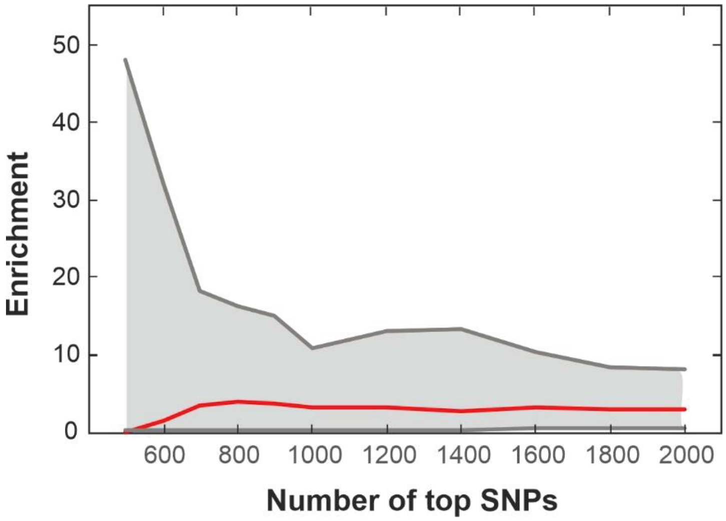

Enrichment of TD21 PGWAS genes in BIO12 EGWAS. The red colored line displays the observed enrichment for different number of top associated SNPs (abscissa), whereas gray color areas show the upper and lower 95% limits of the null distribution. Enrichment is calculated as the ratio between the number of BIO12 top associated SNPs within genes detected for TD21 (±20 kb) and the average number of the null distribution. The null distribution is calculated by 1000 permutations of significance values (Brachi et al., 2010, PLoS Genetics 6, e1000940). TD21: trichome density at 21 °C; BIO12: annual precipitation.

In the following point by point revision we describe the precise changes that we have made in the new version of the manuscript. All changes are highlighted by showing them in yellow color in the two files corresponding to the Main text and the Supplemental Information.

Reviewer 1

General comment

"*This is an interesting study of trichome variation among accessions of Cardamine hirsuta from the Iberian peninsula. The authors provide very interesting data on the diversity of trichome phenotypes in these accessions, the plasticity of these phenotypes in response to temperature, the correlation of trichome phenotypes with environmental variables, and the genes that are likely responsible for the observed variation. The manuscript is well-written and to the point. I do have a few questions, however.*"

We thank the positive view and kind words of Reviewer 1 on our manuscript.

Comments:

1.-"*Most importantly, while I find the identification of specific genes with trichome density and number to be convincing, rounding up the usual suspects plus a few new ones, I am rather less convinced by the GWAS attempting to show a correlation between individual genes and May precipitation in Fig. 4C. While an FDR>0.1 may be a reasonable significance threshold when one has a sharply defined genomic region including many SNPs. But when only a handful of SNPs cross that threshold, and particularly when they are no narrowly focused on a specific genomic region as a distinct peak (as with the GL1 region in 4C), then this seems like a pretty soft threshold.*"

We agree with Reviewer 1 that for some of the regions that we detected by GWAS of climatic variables, only a small number of SNPs show low but significant associations. This is specially the case for the two genes, *ChCLE17* and *ChBOA*, overlapping between the 10 top regions associated with trichome traits and climatic variables, although this is not the case for other environmental GWAS genes of Figure 4C (e.g. chromosome 1). However, as pointed by Reviewer 2 (see point 8), the relevance of our candidate genes associated with May precipitation (EGWAS) derives from their overlapping with the genes detected for trichome traits, and specially for the overlapping with the 10 top genes associated with those traits, as it is the case of *ChCLE17* and *ChBOA* candidates. We think that such overlapping provides an added value in the characterization of trichome associated genes. In addition, given the limitation pointed by Reviewer 1, we also try to support these two regions by showing their opposite effects on trichome traits and May precipitation (Supplemental Figure S8), in agreement with the trichome trait/climate associations (lines 390-393). In the new version of our manuscript, we have emphasized in the Results section (lines 389-390) the limitation pointed by Reviewer 1. Furthermore, in Results and Discussion we have rephrased our conclusions to be very cautious with these candidate genes, by referring to them as "suggestion" (lines 392-393) and "potential candidates" (line 594).

2.-*"Second, I think that insufficient attention was given to the possibility that trichome density variation may be due as much to drift as to selection. Given the frequency with which glabrous varieties have arisen independently in Cardamine, Arabidopsis and related species, I'm not sure that I would describe this as "convergent evolution" (line 530, etc.). It seems to me that the observed variation might be best described by a balance between weak positive selection for trichomes and drift towards loss of function."*

We are aware that the term "convergent evolution" is a fuzzy concept in plant biology, because it is often used in an ambiguous manner to refer to different ideas. For this reason, we carefully define it in the Introduction (lines 127-129) and in Discussion (lines 558-560) according to precise authorized references (Stern, 2013; Morris, 2015). Accordingly, we think that our use of "convergent evolution" is correct because it refers to the independent evolution of a similar phenotype in different species, but it does not necessarily imply a similar role for natural selection. In addition, we agree with the balanced proposition of Reviewer 1 for the observed variation for trichome phenotypes. In our manuscript, we had pointed in Discussion a related complex evolutionary scenario by suggesting "heterogenous selection in fluctuating environments" (lines 601-605). However, following Reviewer 1 suggestion, in the new version we now give more attention to genetic drift by adding in the Discussion the following sentence (lines 605-607): "However, we cannot discard that Brassicaceae diversity for trichome density is the result of a balance between positive selection on hairy phenotypes and genetic drift on *GL1* loss-of-functions." In addition, the possible contribution of genetic drift to trichome diversity has also been added in the conclusion of Results section (lines 431-433).

3.- "*Third, I have to admit that I had a hard time interpreting Fig. 3G and H, and I think this could be explained better for a more general audience. In particular, I didn't understand what is meant by "the 10-15% neighboring populations." It was also not clear to me why the box over these two panels includes both P<0.05 and P>0.05. The significance threshold should be enough, unless I am missing something.*"

We agree with Reviewer 1 that our description of Geographically Weighted Regression (GWR) analyses required additional information for readers that are not familiar with this method. In the current version of the manuscript, we have extended the description of the general method in the Results section to explain better the meaning of "the 10-15% neighboring populations" (lines 321-324). We also agree with Reviewer 1 that the content of the upper box of Figures 3G and 3H was not intuitive and was not properly explained. These boxes show the mean values ± standard deviation of trichome and climate variables for locations with significant or non-significant GWR coefficients (indicated as GWR coeff. $P<0.05$ and $P>0.05$, respectively). The differences between both types of locations for these variables is what we tested statistically. This information has now been added to the legend of Figure 3 (lines 960-962) and Supplemental Figure S5 (lines 249-252). In addition, we have also extended the description of GWR analyses in the Materials and Methods section (lines 706-716).

4.- "*Finally, in discussions of trichome function, I think the authors should cite the Mauricio and Rausher 1997 paper in Evolution, which was the first paper that I am aware of that tested trichome function in a ecology field experiment.*"

We thank to Reviewer 1 for pointing this pioneering and really important reference. This has now been added in the Discussion section (line 603).

Reviewer 2

General comments

1.- "*Excellent background introduction on molecular control, development and natural variation among trichome patterns in plants, including correct use of convergent evolution. I recommend GWAS, rather than GWA studies. And consider P-GWAS for measured traits, and E-GWAS for environments from collection location.*"

We thank the kind words of Reviewer 2. Following his suggestion in the new version of our manuscript we have replaced GWA studies (or analyses) with GWAS, and we have included the use of PGWAS and EGWAS for phenotypic and environmental GWAS, respectively. These acronyms have been changed accordingly throughout the text and in Supplemental Materials.

2.- *"The study sample contains key genetics, trait, and environmental variation enabling dual investigations of how gene variants control quantitative (potentially) adaptive traits AND how gene variants are controlled by, or filtered by, the environment. When this is done in multiple species, discoveries like "opposite climatic associations than A. thaliana, supporting that this trait might be involved in partly distinct environmental adaptations in the two plants." This is rather unexpected for adaptive traits in related (ecologically swappable) species. Could it be due to differential geographic structure? Might another there be another environment (water vs temp?) with stronger effect that is differentially correlated with trichomes? This is not easy to test, perhaps some permutations of traits and environments with locations could be a type of null hypothesis [this is outside the scope of this paper but just something for the authors to consider]".*

We agree with Reviewer 2 that the opposite climatic associations observed in *C. hirsuta* compared to *A. thaliana* is one of the most interesting and unexpected results of our study. We also agree with Reviewer 2 that other environmental factors correlating with our climatic variables might contribute and that they might interact with the genetic background. This complexity is addressed in Discussion (lines 475-489) where we describe the differences between both genetic groups. However, in the new version of our manuscript we have further emphasized the complexity of correlated traits by adding the following sentence (lines 541-543): "Nevertheless, we cannot exclude that other environmental factors correlating with climatic variables might drive the differential evolution of trichome pattern in these species."

Technical issues.

1.- *"123 wild accessions is not a small sample side, but pretty good considering they from different locations? What do bioclim PCs look like for this sample set? Much spread in environmental space?"*

Following the suggestion of Reviewer 2, we have now carried out PC analyses to illustrate the broad environmental space covered by *C. hirsuta* populations in the

Iberian Peninsula, as well as the environmental differentiation between the two genetic groups identified in this region. In addition, we have also generated density plots of the seven environmental variables used for the distribution models, which show better the differential environmental distributions of both genetic groups (IBE and BAL). This information has been described in Supplemental Methods (lines 15-18 and 26-28), and it has been included in a new Supplemental Figure (Supplemental Figure S3), which is also mentioned in the Main text (lines 203-204). We believe that this additional information will also reinforce and facilitate the interpretation of our results on species distribution models, as pointed below by Reviewer 2 (see point 4) and Reviewer 3 (see point 5).

2.-"*Both analyses found two highly diverse and differentiated genetic 2 groups (FST=0.23..) is rather high, but not unusual for inbreeding lines.. This is considered a deeper speciation levels of separation in largely outcrossing species, where we wouldn't expect structure to be able to accommodate levels of differentiation.*"

We agree with reviewer that for outcrossing species this might be an excess of differentiation. However, *C. hirsuta* is an autogamous plant, and therefore it can display larger differentiation. As pointed by Reviewer 3 (see minor point 1) the mating system of *C. hirsuta* was not indicated in our manuscript, but this information has now been included in the Introduction (line 137).

3.1.- *"Fig1 is a huge and strong dataset! IBD [panels DEF] assub geographic distance is linear in km, but there is certainly isolation by mountains. The authors might consider rather geographic resistance surfaces, that extend the very beautiful environmental niche mappings among subspecies (Fig1H).*

We find the suggestion of analysing the isolation by resistance (IBR) as complement for the isolation by distance (IBD) interesting but, as shown in a previous study from our laboratory in this same region, this analysis displays limited power due to its complexity. In particular, as we have previously shown for *Arabidopsis thaliana* in the Iberian Peninsula (Castilla et al. 2020; BMC Evolutionary Biology 20:71) IBR displayed lower explanatory power than IBD and IBE (isolation by environment). In addition, due to the multiple correlations among resistance and environmental variables, addressing IBR requires careful and deep comparisons with IBE to determine the proper environmental niche. For this reason, we think that the environmental dissection of the ecological niche of *C. hirsuta* and the genetic groups is more precisely addressed by applying potential distribution modeling (as we have done in Figure 1G-1J), since this method is focused on specific environmental factors.

3.2.-"In Fig1F, the 2 groups of pink points, I worry about the 5 BAL populations -140 Eigenvector2 in Fig1B. Where are they on the map? New studies could expand on this region for sampling, as well as the admixed populations. I would like to see a population sample dendrogram just to identify and likely remove 1 of a pair of closely related individuals, or recent hybrids, eg 4 pairs with 0 genetic distance are not genetically distinct individual samples, < 5% might be recently hybrids not adding

resolution to GWAS. Slightly reducing this sample size won't hurt association mapping as these samples are not independent, but will the reduce bias they would create."

Following the suggestion of Reviewer 2, we have now included a new Supplemental Figure S1B and S1C showing the geographic distribution of the six BAL accessions with low PC 2 values. As shown in that figure, those accessions are distributed across Iberia, with no clear geographic pattern, which indicates that they are not associated to a particular region. This additional diversity of BAL group has now been described in the Main text (lines 184-187). In addition, following also the suggestion of Reviewer 2, we have now analyzed the genetic structure of the 123 accession by neighbor-joining (NJ) clustering analysis, and this information has been described in the Main text (lines 174-175; line 739), Supplemental Methods (lines 78-85 and 110-111) and as a dendogram in the new Supplemental Figure S1A. This analysis shows that only four pairs of accessions have genetic distances between 0.05 and 0.001, and only one of these pairs belongs to the six BAL PC 2 differentiated accessions. We have also described now the geographic distribution of these four pairs of accessions (Supplemental Figure S1C; lines 85-87 of Supplemental Methods), showing that again they have no geographic pattern because these pairs appear at geographic distances from 2 to 512 km. As pointed by Reviewer 2, we understand that maintaining those accessions (genetically non-independent samples) might increase certain bias for PGWAS. However, given the geographic and environmental dispersion of each of these pairs of accessions, we think that removing some of these samples might also create a bias in environmental comparisons in unknown directions. We want to emphasize that all methods that we have used for GWAS and environmental analyses take into account the lack of independence among samples and aim to correct it through the kinship matrix (PGWAS) or autoregression coefficients (environmental analyses). Taking into account these considerations (the small number of closely related accessions; their geographic dispersion; the statistical methods used to take into account genetic and environmental correlations among samples), we prefer to maintain the complete dataset for all analyses, but adding this relevant genetic and geographic information in the manuscript. In this way, we believe that our study better illustrates the complexity and limitations of comparing large collections of populations from highly diverse species at environmental and genetic levels. To this end, in the current version of the manuscript, we have added the new Supplemental Figure 1S including the dendogram of the 123 samples (Figure S1A), and the geographic distribution of pairs of closely related accessions (Figure S1C). The relatedness between accessions and the NJ clustering analyses have now been described in the Main text (lines 184-187) and in Supplemental Methods (lines 83-87).

4.- *"I would show the 123 environmental points from sampling locations on Fig 1 I & J, as it looks a bit too smooth. The distribution of Agriculture % is very interesting. How was this calculated? %Ag/km^2, is that annual cropped land only? Or are perennial pastures included? Could you add the % var that each bioclim +Ag contributed to suitability color in Fig 1 G&H?"*

As indicated in point 1 of Reviewer 2 and point 5 of Reviewer 3, we realized that more information on species distribution models is necessary to avoid doubts and potential misunderstanding. We have now indicated in the legend of Figure 1 (line 918) and in Material and Methods section (line 683) that distribution models are continuous, since they are based in estimates of suitability using 1 km grid. However, following Reviewer 2 suggestion, we have added *C. hirsuta* locations used for the generation of distribution

models in Fig 1G and 1H and in the corresponding legend (lines 921-923). Regarding the contribution of the seven environmental variables to the model, this information was already provided in Supplemental Table S4, but now it has been also added in Figures 1I and 1J, and this has been explained in the corresponding legends (lines 925-927) and in Supplemental Methods (lines 39-42). Finally, the variable %Agriculture was calculated by counting the number of 100 m grids belonging to any of the 11 agricultural categories described in CORINE Land Cover, which include arable land, permanent crops pastures and heterogeneous agricultural areas. This information has now been added in Materials and Methods (lines 638-641).

5.- *"4.5M SNPs is a relatively large number? What is the extent of LD? [first remove related samples]. Fig 5b, looks like an extensive LD region over 60kb of elevated associated SNPs, this is difficult to confirm a gene, but also a larger target for trait-environmental selection, eg supergene."*

The nucleotide diversity and linkage disequilibrium of *C. hirsuta* has been previously described in Baumgarten et al., 2023 (PLOS Biology 21: e3002191) through the analysis of the genome sequences of 488 European accessions, including 48 Iberian accessions in common with this study (as indicated in our manuscript in lines 180-183 and 626-628). Baumgarten et al., (2023) showed that nucleotide diversity of *C. hirsuta* is about twice that of *A. thaliana*, which explains the large number of polymorphisms found in our new study. Baumgarten et al., (2023) also showed a detailed comparative analysis of LD between *C. hirsuta* and *A. thaliana* and in different genetic groups (IBE and BAL included) carried out with PopLDdecay (Zang et al., 2019, Bioinformatics 35: 1786-88) excluding closely related accession and pericentromeric regions. This study shows that average LD of *C. hirsuta* in IBE and BAL groups extends up to 20 kb. Therefore, in agreement with Reviewer 2 comment, the LD in in *ChGL1* region of Figure 5B is larger than average. This information has now been added in the Results section of the new manuscript (lines 412-414).

6.- "*Fig 2 is equally powerful, true multitrait developmental phenomics approach! Ontogeny is so important and difficult to consider. Trichome number and size of largest leaflet were used to determine trichome density (density has lower heritability), but trichome number and leaflet size may covary and be relate with genotype. Can you show a scatter plot of trichome number and leaf size for density and consider a residual trait trichome number per size expected deviation rather than a density ratio? You might have more power with using leaflet size as a covariate for trichome number than trichome density, but the results are very clear and these are related traits identifying the same genes in practice. Table1 shows overall very high heritability and major loci effects beyond kinship (that was not unexpectedly large given major structure groups).*"

Following the suggestion from Reviewer 2 we have made a scatter plot showing the relationship between trichome number and leaflet size at 21 and 26 °C (see new Supplemental Figure S4). As shown in those plots, both variables only correlate significantly at 26 but not at 21 °C, in agreement with the correlation values shown in Supplemental Table S6. Therefore, the suggested new variable measuring the expected deviations might only work for one temperature but not for the other. In addition, we think that trichome density is more intuitive to compare with the remaining variables, and, therefore, we have kept our measurements of trichome density.

Nevertheless, in the new version of the manuscripts, the plots showing the relationship between trichome number and leaflet size or trichome density have now been included as part of a new Supplemental Figure S4.

7.- *"The plasticity ratio isn't great statistically. Best to fit GxE for QTL-SNPs using measured data. This isn't critical however."*

We agree with Reviewer 2 that plasticity ratios have low statistical power. However, we think that, for readers not familiar with GxE interactions, these measurements of plasticity are more intuitive and, as shown in our study, still allow detection of significant candidate loci that might explain GxE interaction. In addition, we believe that carrying additional GWAS analyses based on SNPxE interactions will require additional complex comparisons that will extend the manuscript beyond its scope. Nevertheless, following the suggestion of Reviewer 2, we have now tested the SNPxE interaction for the most significantly associated SNPs, which supports the potential contribution of some of our candidate genes to explain the genotype by environment interaction found for trichome traits. This information has now been added in Results (lines 371-377) and Materials and Methods (lines 775-778), as well as in Supplemental Table S8.

8.- *"Testing the SNP x climate variation and SNP x trichome trait variation in GWAS is a super powerful way to get at the causal trait and environment links. There is some overlap with genes identified in both scans, suggesting the May precip variable as linked with trichome genetics. I didn't see a formal enrichment test if SNPs were more common than expected, but they are likely to be. Eg 17 genes in 11 regions overlap."*

Following the suggestion of Reviewer 2, we have now carried out a formal test to measure gene enrichment, using an R script previously described (Brachi et al., 2010, PLoS Genetics 6, e1000940; Tabas-Madrid et al., 2018, Plant Cell & Env. 41, 1806-1820). As example, here we are including a Figure for Reviewers with the results of the test for enrichment of TD21 PGWA genes in BIO12 EGWA analysis (see Figure for Reviewers.doc). In brief, enrichment is calculated as the ratio between the number of BIO12 top associated SNPs within the genes detected for TD21 (±20 kb), and the average number of the null distribution. The null distribution is calculated from 1000 permutations. As shown in this Figure, there is no significant enrichment of PGWA genes in EGWA analyses. We think that the expectation of gene enrichment suggested by Reviewer 2 derives from an unclear sentence in the Results section of our manuscript, suggesting that the 17 overlapping genes are detected in each PGWA/EGWA comparison. However, these 17 genes correspond to the total number of genes identified by overlapping all trichome variables and the three environmental variables. We have now rephrased this sentence to avoid misinterpretation (lines 381-383). In addition, we consider that including these formal enrichment tests in the new version of our study will mainly distract readers from the main message and will enlarge a manuscript that is already rather long. Nevertheless, if the Editor or Reviewers consider it convenient, we will add them as another Supplemental Figure.

9.-"Distinct environmental trichome responses for A thaliana vs C hirsuta is intriguing, a caveat being the focus on Col and Ler strains of A thaliana. Might different responses

to seasonal precipitation be related to seasonality of flowering? Does C hirsuta also have rapid cycling stage?"

We agree with Reviewer 2 that the different environmental responses of *A. thaliana* and *C. hirsuta* across development (ontogeny), specially in the abaxial leaf surface, is an interesting and intriguing result. However, as pointed in our Discussion (lines 507-509), it requires the analysis of *A. thaliana* accessions other than Col and L*er*. Regarding Reviewer 2 question on the rapid cycling in *C. hirsuta*, like in *A. thaliana*, there are some rapid cycling varieties, which have been shown to be caused also by *FRI* loss-of-function alleles (Baumgarten et al., 2023). As it has been shown in *A. thaliana* (see Wang et al., 2019, The EMBO Journal 38: e100063 cited in our Introduction), some flowering genes might affect trichome density through their effect on juvenility. However, such genes show rather small effect on trichome traits and did not appear in association studies for trichome pattern in *A. thaliana* (Atwell et al., 2010; Arteaga et al., 2022), or in our study of *C. hirsuta*. Therefore, it is unlikely that the different trichome responses described in these species are related with flowering variation, although specific studies addressing both traits simultaneously are needed.

10.-*"This is a very powerful, well designed and elegant study, covering all the genotype, phenotype and environmental basis to discovery new genes and novel alleles of known genes allowing comparisons among species. It is worthy of a top tier journal!".*

We thank the kind comments and useful suggestions from Reviewer 2, and hope that he will appreciate our extensive and careful modifications and explanations.

Reviewer 3.

General comment

"The authors established a new genetic data set of Cardamine hirsuta populations in the Iberian Peninsula and explored local adaptation signatures, primarily focusing on leaf-related phenotypes, including trichome numbers. They conducted comprehensive analyses of environmental adaptation utilizing climate and genetic data, and compared adaptation features between species at the population level. The results demonstrated convergent evolution in trichome density, but also independent environmental adaptations are going on. Their approach delves into the deeper evolutionary processes that comparative genomics alone cannot fully reach."

We thank the positive view of Reviewer 3 on our manuscript.

Comments:

1.-"*I have high expectations for the potential of this approach; however, I'm somewhat disappointed by what appears to be a superficial comparison of the evolutionary processes related to the genetic architecture underlying trichome number/density adaptation. As mentioned in the abstract, "the degree of parallelism in the genetic mechanisms underlying this diversity and its environmental drivers in different species remains unclear." Hence, a demonstration of parallel changes in genetic basis is*

crucial, at least through the detailed comparison to A. thaliana (i.e., genetic structure, allelic distribution, and natural selection).”

We understand that the limited comparison between the two species pointed by Reviewer 3 derives, on one hand, from the fact that *A. thaliana* trichome diversity has been previously described in a separate study and, therefore, in this study we focus our experimentation only on *C. hirsuta*. Despite this limitation, most of the discussion of our manuscript is focused on the comparison between *C. hirsuta* and *A. thaliana*. In particular, the first section of Discussion compares in detail the genetic structure of both species at global and regional scales. Similarly, the second and third sections concentrate on the interspecific comparison of the environmental and genetic mechanisms accounting for the diversity for trichome patterning, respectively. We are aware that our comparisons of *C. hirsuta* and *A. thaliana* are mostly based on two separate studies in the Iberian Peninsula, which demands certain assumptions on our previous *A. thaliana* study. To facilitate the comparison of both species, following Reviewer 3 suggestion, in the new version of our manuscript we have included a new Supplemental Figure S2 comparing the genetic and geographic structures of *C. hirsuta* and *A. thaliana* in the Iberian Peninsula. This Figure and the parallel structure of both species is now included in Results section (lines 188-190) and Discussion (line 460). This figure has been added as supplemental figure because the Results section is exclusively focused on *C. hirsuta* and including a panel with *A. thaliana* will largely disrupt the structure of the manuscript.

On the other hand, we think that the limited number of genes currently identified in each species as contributing to trichome variation, together with their low allelic frequency and the scarce knowledge on the evolutionary mechanisms shaping this diversity, also limit a deeper comparison between *C. hirsuta* and *A. thaliana*. In this respect, we agree with point 6 of Reviewer 3 and, following his/her suggestions, we have now added another Supplemental Figure S9 comparing the genetic and geographic diversity of *GL1* to support the parallel evolution of this gene in both species (see point 6). Overall, despite these limitations, we believe that our study provides one of the most complete comparative analyses of the intraspecific diversity for trichome pattern in two plant species, including the three sources of variation (phenotypes, environments, and genotypes) that are necessary for evolution. We are also aware that this is just a first step, and a deeper knowledge will surely be achieved in future studies comparing specific genes in both species, specially when segregating in the same geographic locations.

2.-*“Understanding environmental adaptation is the main topic of this study, and Fig 3B highlights strong genetic environment interaction (G x E) in all traits. The dissection of loci related to GxE may help in interpreting the genetic architecture and the geographic distribution of alleles. One related study in A. thaliana is below. They analyzed G x E in FLC using GWAS. Sasaki et al., (2015) "Missing" G x E Variation Controls Flowering Time in Arabidopsis thaliana. PLOS Genetics 11(10): e1005597.”*

We agree with Reviewer 3 that identifying the loci contributing to G x E interaction will help the interpretation and understanding of the genetic architecture. However as indicated by Sasaki study, finding those loci “has proven extremely challenging” and several methods have been proposed for such analyses, including Sasaki’s method, as well as the method indicated by Reviewer 2 in point 7. As discussed in point 7 of Reviewer 2, we believe that carrying additional GWAS analyses specific for G x E will

require in depth comparisons and will extend a manuscript already rather dense beyond its scope. However, as also described in point 7 of Reviewer 2, to support the contribution of some of our candidate genes to the G x E interaction, we have now tested SNP x E interactions for the most significantly associated SNPs. This information has been added in the Main text of our manuscript (lines 267-268, lines 371-377, lines 775-778) and in Supplemental Table S8, to emphasize the relevance of G x E interaction and the complex underlying genetic architecture.

3.- "*Regarding the genetic architecture, how much phenotypic variation can be explained by ChGL1 genetic variation? The expression of ChGL1 (cis-regulation?) explains a trichome density of more than 80%. But what is the cause of the large expression variation? (Fig 5 E&F). This scenario looks similar to the regulation of flowering time by FLC in A. thaliana population. Are the identified GWAS peaks associated with multiple haplotypes, is the ChGL1 expression regulated by other alleles identified in GWAS, or genetic background (polygenic)?"*

ChGL1 explains 19 to 27% of the phenotypic variance for TN and TD traits in the 118 non-glabrous accessions. The expression of *ChGL1* accounts for 80% of the variance for trichome density, but in the set of 12 accessions analysed for gene expression. This information was absent in our previous version, but it has now been added in the Results section (lines 419-422; lines 424 and 427). As indicated by Reviewer 3, this scenario resembles the regulation of flowering time *in A. thaliana*, where several independent mutations affect the function of *FLC*. However, in our case, when excluding the null alleles (glabrous accessions) we only found one haplogroup (the group of 10 haplotypes differentiated in Figure 5C and so-called PGWAS haplogroup) associated with trichome traits. The differences in *ChGL1* expression between accessions of this haplogroup and the rest of accessions are explained by one or several of the 23 SNPs detected in *ChGL1* region, or by some other cis-regulatory polymorphisms in linkage disequilibrium with them, which must be present in the 10 accessions belonging to this haplogroup. This information was probably described in a confused way in our previous version, but to avoid misinterpretation we have now rephrased lines 421-423.

4.- *"For Fig 1A, the explanation of why the authors used K=2 (from their previous Pan-genome paper?) is needed. Also, in the map, which lines are admixture that the Balkan group introgressed?"*

The final K=2 value was estimated as described previously by the authors of the model-based clustering algorithm implemented in ADMIXTURE (Alexander et al., 2009). Basically, the most likely K value was estimated as that showing the lowest cross-validation error. This information has now been added in Supplemental Methods (lines 92-97). In addition, we have included a new Supplemental Figure S1D, showing the change in the cross validation errors across the different K values tested. Furthermore, following the suggestion of Reviewer 2 (see point 3.2) the two genetic groups found for *C. hirsuta* in Iberia have now been also supported with the analysis of genetic structure by the neighbour-joining (NJ) clustering method. This information has been now described in the Main text (lines 184-186), in Supplemental Methods (lines 78-87 and 110-111) and in the new Supplemental Figure S1A.

With respect to the “admixture lines” mentioned in this point by Reviewer 3, we are not sure what is meant because Admixture, PC and NJ analyses did not show admixed individuals (all accessions are almost fully assigned to IBE or BAL group). We understand that Reviewer 3 refers to the same question of Reviewer 2 (see point 3.2) on the additional diversity found within BAL group by the principal component analysis. In the new version of the manuscript, we have now described this BAL diversity in the Main text (lines 184-187) and we described the geographic pattern of those BAL accessions in the new Supplemental Figures S1B and S1C.

5.- “*For Fig I &J, if my understanding of the model is correct, the suitability of Bio1 in all populations appears to be almost the average of the two populations. However, the observed pattern differs significantly from both. Could the authors provide an explanation for this inconsistency?”*

As pointed also by Reviewer 2 in points 1 and 4, we realized that we should provide more information on species distribution models. We have now explained the meaning of predicted suitability and suitability response curves in Materials and Methods (lines 687-693) and in the legend of Figure 1 (lines 919-920). As described therein, the predicted suitability is a relative index of adequacy of the species, or the genetic groups, to the environment where it occurs. However this is not meant to be read as absolute probabilities of presence, but as how suitable a given location or grid cell is with respect to the others. The predicted suitability and response curves of each model depend on numerous factors, including the number of locations of the species used for the generation of the model, as well as the relative contribution of each environmental variable. Both parameters have now been included in Figures 1G and 1J (lines 921-923 and 925-927 in the legend of Figure 1), and they explain why the response curve of the species is related to the curves of the two genetic groups but is not the direct average of them.

6.- “*If the authors could superimpose the allelic distributions of A. thaliana causing changes in trichome density phenotypes in Fig 5 A and D (or just add next to the panel for C. hirsuta), it could help the interpretation of the "parallel evolutionary process" of adaptive traits on a fine scale.”*

Following the suggestion of Reviewer 3, we have included a new Supplemental Figure S9 showing the different functional alleles (complete and partial loss- and gain-of functions) described for *GL1* in *C. hirsuta* (this study) and *A. thaliana* (previous studies; Arteaga et al., 2021, 2022) in Iberia. This figure includes the location of loss-of-function mutations in similar gene regions in both species, as well as the geographic distribution of the various alleles in the Iberian Peninsula. This information is now described in the Results section (lines 401-403) and is also pointed in Discussion (line 574) to support the parallel evolution of *GL1* in both species. This figure has been included as supplemental figure because, as mentioned in point 1, the Results section and Figures are exclusively focused on *C. hirsuta*, and we think that adding a panel with *A. thaliana* in Figure 5 will largely disrupt the structure of the manuscript.

7.- “*L369: The author mentioned that ChCLE17 and ChBOA alleles were also associated with the precipitation. Are the alleles identical between GWAS for the phenotype and climate variables*?”

The SNPs showing the strongest associations for PGWAS and EGWAS were different in both analyses, although several SNPs appeared as significantly associated in both variables. As indicated in the Materials and Methods section of our manuscript, “Given the complexity of quantitative traits and the limited population size, comparisons of GWAS results among different variables are based on the overlapping of genes instead of SNPs”. This has now been rephrased in Materiasl and Methods (lines 762-764) to make it clearer.

8.- “*L404 I didn't get the reason clearly why the authors concluded it. Could the authors explain in detail*?”

We agree with Reviewer 3 that this sentence was rather cryptic. We have now extended our conclusion and the rationale behind it (lines 431-433 of new version).

<u>Minor comments:</u>

1.- *“L136-137: Including information about the mating system (self-pollination) would be helpful for readers.”*

We agree with Reviewer 3 that this information is important and we have now added this in line 137.

2.- *“L188-190: Could you rephrase this part? It is unclear what the authors mean.”*

This sentence has been rephrased to make it clearer (lines 197-198 in the new manuscript).

3.-*“Fig1 I & J: Please add explanations of 'suitability' in the main text and M&M”.*

As described in point 5 of Reviewer 3, this information has now been added in Materials and Methods (lines 687-693) and in the legend of Figure 1 (lines 919-920). In addition, information on distribution models has also been extended in Supplemental Methods (lines 39-47).

4.- *“L220: To me, the peak seems at "four leaves" rather than "first five leaves" in Fig 2C”.*

We agree with Reviewer 3 that the maximum peak is on leaf four. However, for some accessions (Loz-12, Hin-0 and Slc-0) the value in leaf five is not significantly different from that at leaf four and, therefore, we think that is more rigourous to keep the peak on both leaves. Accordingly, we have rephrased this sentence in line 228 of the new version as: “…whereas terminal leaflet size increased only in the first four to five leaves…”.

5.- *“L538: It might be valuable to include a citation for the recent study. Xuan et al., Xuan, Lijie, et al. "Genome-wide association study reveals new genes involved in leaf trichome formation in polyploid oilseed rape (Brassica napus L.)." Plant, Cell & Environment 43.3 (2020): 675-691.”*

We had missed this reference and thank the Reviewer for pointing to it. We have now cited this this reference (lines 112, 567, and 604) and added in the reference list.

25-Mar-2024

Dr. Carlos Alonso-Blanco
Consejo Superior de Investigaciones Cientificas
Plant Molecular Genetics
Madrid, Sp N/A
Spain

RE: Trichome pattern diversity of Cardamine shares genetic mechanisms with Arabidopsis but differs in environmental drivers

Dear Dr. Carlos Alonso-Blanco:

We are pleased to accept your manuscript for publication in the Plant Physiology. This acceptance is contingent on revision based on the comments of our reviewers. In particular, please consider incorporating the comments suggested by Reviewer3 to improve clarity of the manuscript.

Please highlight all changes and include a detailed annotation to changes to the text, with line numbers, and noting your responses to the comments.

To submit your revised manuscript, click:

Link Not Available

If you cannot return the revised manuscript within 8 weeks of receipt, please let us know. Otherwise, we will assume that you have elected not to revise the manuscript and are withdrawing it.

Thank you for allowing us to review your work. We look forward to hearing from you soon.

Sincerely,

Board of Editors, Plant Physiology

-- Reviewer comments:

Reviewer #1 (Comments for the Author):

The authors have more than adequately addressed my comments by adding a few additional qualifying statements, particularly on the potential role of drift, which clarifies their definition of convergent evolution. As I am not the most qualified reviewer to address details of the statistics, I have no further comments.

Reviewer #3 (Comments for the Author):

This manuscript has been significantly improved and meets the reviewers' requests satisfactorily. I have a couple of minor comments.

The terminology of PGWAS sounds strange. GWAS for phenotypes is fundamental, so referring to it as PGWAS might cause confusion as it implies a more specific approach.

L197-198, Fig. 1F simply suggests that the BAL group contains two genetically separated populations. Therefore, I don't understand why the authors conclude that the IBE genetic lineage has a more ancient history.

In the following point by point revision, we describe the precise changes that we have made in the new version of the manuscript. All changes are highlighted in yellow color in the file corresponding to the Main text and the Supplemental Information.

Reviewer 3

- *"The terminology of PGWAS sounds strange. GWAS for phenotypes is fundamental, so referring to it as PGWAS might cause confusion as it implies a more specific approach."*

We agree with Reviewer. We added the PGWAS acronym following the suggestion of Reviewer 2, but we are now aware of the new confusing possibility. Following Reviewer 3 suggestion, we have removed the use of PGWAS throughout the manuscript, including Supplemental Information.

- *"L197-198, Fig. 1F simply suggests that the BAL group contains two genetically separated populations. Therefore, I don't understand why the authors conclude that the IBE genetic lineage has a more ancient history."*

We believe that the author has focused only on Figure 1F, where we agree with Reviewer that the two BAL genetic groups described in previous lines of the results section (lines 183-185) are clearly visible. However, in lines 197-198 we refer to the comparison of Figures 1E and 1F. When comparing both panels, the IBD correlation between genetic distance and geographic distance (r value of panels 1E and 1F) is larger for IBE group (r=0.58) than for BAL group (r=0.45). This difference is even larger when the group of six BAL accessions that is differentiated from the rest of BAL accessions is removed, because then the IBD correlation of BAL group drops to 0.38. This larger r correlation value of IBE group than BAL group is the reason to suggest a more ancient history of this lineage. We think that it is not necessary to add this detailed numeric description, because the distribution of points in panel 1F clearly shows that the correlation (IBD r value) of BAL group including all 51 accessions is larger (higher slope) than that of BAL group when considering only the 45 accessions or the 6 accessions (each BAL population).

To avoid misunderstanding we have now rephrased this sentence (lines 197-198) and the previous one (lines 194-196), and moved the correlation values to the second confusing sentence (lines 197-198).

- Finally, we would like to take the suggestions of Reviewer 3 to extend one sentence in relation to point 1 of previous version where Reviewer 3 indicated: "*As mentioned in the abstract, "the degree of parallelism in the genetic mechanisms underlying this diversity and its environmental drivers in different species remains unclear." Hence, a demonstration of parallel changes in genetic basis is crucial, at least through the detailed comparison to A. thaliana (i.e., genetic structure, allelic distribution, and natural selection).*"

To reinforce even more the parallelism between Arabidopsis and Cardamine in the evolution of *GL1* mutations we have extended a sentence in results section (lines 257-

258) indicating the similar frequency of null mutations observed in both species. The sentence extended is: "*and at a similar low frequency of 0.04 than in A. thaliana in this region (Arteaga et al., 2022)*". We believe, that this small added sentence emphasizes a similar frequency of null mutations in both species that otherwise might be missed by most readers.

29-Mar-2024
Dr. Carlos Alonso-Blanco
Consejo Superior de Investigaciones Cientificas
Plant Molecular Genetics
Madrid, Sp N/A
Spain

MSID: PP2024-RA-00219DR1
MS TITLE: Trichome pattern diversity of Cardamine shares genetic mechanisms with Arabidopsis but differs in environmental drivers

Dear Dr. Carlos Alonso-Blanco:

I am pleased to inform you that your revised manuscript "Trichome pattern diversity of Cardamine shares genetic mechanisms with Arabidopsis but differs in environmental drivers" has been accepted for publication in Plant Physiology. Your manuscript will be checked for consistency with journal formatting and image standards by one of our Science Editors, who will contact you shortly. They may have a number of queries and suggestions for you. Please keep in mind that they work to increase the online visibility of your article and its attraction for readers from the widest of backgrounds. You will have the opportunity to review any revisions, and I hope you will work constructively with them to get the most out of your research. You will also have the opportunity to review all changes when you receive the author proofs for your article.

Once validated for publication, your article will be sent to a Science Editor for a final review of your manuscript and source files. After your final files are uploaded, your article will appear in the next available issue and the online Preview version of your article will be posted after your signed Copyright and Checklist forms and final source files are received.

Please be sure to include the following in your manuscript file:
Harvard style citations
List of all author contributions and funding information at the beginning of manuscript after the titles and author list.
List all supplemental materials associated with the article, with titles, at the end of the ms, before the references and acknowledgments section.
Complete Figure Legends for all figures appearing in the article listed before the Literature Cited.

Thank you and congratulations on your Plant Physiology paper!

Sincerely,
Board of Editors, Plant Physiology

=====
FIRST AUTHOR PROFILES
=====

We recognize the hard work that goes into being First Author of a Plant Physiology paper by publishing a "First Author Feature" for each First Author alongside your paper.

If the first author(s) would like to submit information for a feature on Facebook and Twitter, fill out the form by clicking on the link below:

Plant Physiology First Author Feature Form: https://forms.office.com/r/ZBCGZfhm8F
If you cannot access the form through the link above, please contact Rachel at rbelsky@aspb.org.

To upload an image of yourself to accompany your profile, please put your photo in the correct journal folder at the link below. You must label your image: Author First Name Last Name Article Title.

First Author Profile Image Dropbox: https://www.dropbox.com/scl/fo/xz029p7qjr2jml2rp2kmq/h?dl=0&rlkey=6fkxcaqmxx35ap152b2osy1fz

=====
IMPORTANT REMINDER: PEER REVIEW REPORTS
=====

If you opted to publish a peer review report along with your article during the original submission process, it will be prepared by the editorial staff and publicly posted with your manuscript, inside the zip file that contains any other supplemental material. As a reminder, the peer review report is a public record of all comments from editors and reviewers, as well as your prior responses, as you received them in the decision letters for each draft of your manuscript. If you agreed to publish this report and have changed your mind, or are not sure if you selected this option, please contact the editorial office as soon as possible before signing the license agreement from our publisher.

-- Reviewer comments:
